# Protective effects and mechanisms of puerarin in ovariectomized animal models of osteoporosis: a systematic review and meta-analysis of preclinical studies

**DOI:** 10.3389/fphys.2026.1911109

**Published:** 2026-08-27

**Authors:** Chao Dong, Zhixun Guo, An Hu, Liyang Zhao, Ying Tang, Yexin Chen, Zhichao Ruan, Yixin Ma, Jiangteng Liu, Xinyu Wang

**Affiliations:** 1 Beijing First Hospital of Integrated Chinese and Western Medicine, Beijing, China; 2 Dongzhimen Hospital, Beijing University of Chinese Medicine, Beijing, China; 3 Beijing University of Chinese Medicine, Beijing, China; 4 The First Affiliated Hospital of Zhejiang Chinese Medical University (Zhejiang Provincial Hospital of Chinese Medicine), Hangzhou, Zhejiang, China; 5 Shaoxing People’s Hospital, Shaoxing, Zhejiang, China; 6 School of Medicine, Shaoxing University, Zhejiang, China

**Keywords:** animal experiment, meta-analysis, osteoporosis, puerarin, systematic review

## Abstract

**Background:**

Osteoporosis is a prevalent systemic metabolic disease, and osteoporotic fractures severely compromise patients’ health and quality of life. Postmenopausal osteoporosis is a major form of primary osteoporosis in women and is primarily attributable to estrogen deficiency. Ovariectomized animal models are widely used to reproduce estrogen deficiency–induced bone loss and microarchitectural deterioration. Puerarin, a natural isoflavone extracted from the traditional Chinese herb *Pueraria lobata* (kudzu), exhibits antioxidant, anti-inflammatory, and bone-metabolism-modulating pharmacological properties. Although some studies have conducted meta-analyses of puerarin in rat models of osteoporosis, there is a lack of analysis on bone microarchitecture and mechanism-related indicators.

**Objective:**

This systematic review and meta-analysis aimed to evaluate the effects of puerarin in female ovariectomized animal models of osteoporosis and to synthesize the potential mechanisms underlying its bone-protective effects.

**Methods:**

Seven databases (PubMed, Web of Science, Embase, CBM, CNKI, WanFang, and VIP) were systematically searched. The methodological quality of included studies was assessed using the SYRCLE risk-of-bias tool. Statistical analyses were performed with STATA 14.0. Primary outcomes included bone mineral density (BMD), trabecular number (Tb.N), trabecular separation (Tb.Sp), trabecular thickness (Tb.Th), bone volume fraction (BV/TV), and connectivity density (Conn.D). Secondary outcomes encompassed bone metabolism markers, inflammatory cytokines, and oxidative stress indices. Sensitivity analyses and subgroup analyses were conducted to explore sources of heterogeneity. Publication bias was evaluated using funnel plots and Egger’s test.

**Results:**

A total of 22 studies comprising 447 animals were included in this review, and all osteoporotic models were constructed via bilateral ovariectomy. Puerarin significantly improved BMD, Tb.N, Tb.Th, BV/TV, and Conn.D, and reduced Tb.Sp. For secondary outcomes, puerarin significantly decreased serum levels of bone turnover markers (BGP, PINP, CTX-I, TRACP-5b, and RANKL) and increased OPG levels. Additionally, puerarin significantly reduced pro-inflammatory cytokines (IL-6, TNF-α) and malondialdehyde (MDA), while elevating antioxidant enzyme activities (SOD, GSH-Px).

**Conclusion:**

Puerarin showed protective effects in female ovariectomized animal models of osteoporosis, potentially through suppression of bone resorption, normalization of high bone turnover, and anti-inflammatory and antioxidant actions. Because the evidence was heterogeneous and limited to ovariectomy models, further rigorous preclinical studies are required before clinical translation.

**Systematic review registration:**

https://www.crd.york.ac.uk/PROSPERO/, identifier CRD420251236043.

## 1 Introduction

Osteoporosis is a common systemic metabolic bone disease characterized by reduced bone mass, deterioration of bone microarchitecture, and increased bone fragility, clinically manifesting as elevated fracture risk (Consensus development conference: diagnosis, prophylaxis, and treatment of osteoporosis, 1993). Based on etiology, osteoporosis can be classified into primary and secondary types. Primary osteoporosis is further categorized into three subtypes: postmenopausal osteoporosis (type I), senile osteoporosis (type II), and idiopathic osteoporosis (juvenile form). A 2021 meta-analysis estimated that nearly one-fifth of the global population is affected by osteoporosis, with a prevalence of 23.1% in women and 11.7% in men (Salari et al., 2021). Epidemiological surveys indicate that among individuals aged 50 years and older, 43.1% have low bone mass and 12.6% meet the diagnostic criteria for osteoporosis, with the prevalence of both low bone mass and osteoporosis being higher in women than in men (Sarafrazi et al., 2021). As population aging accelerates, the number of osteoporosis patients continues to rise. Osteoporotic fracture, the most severe complication of osteoporosis, substantially impairs quality of life, and the prolonged immobilization following fracture often leads to complications with high disability and mortality rates, imposing a heavy burden on both society and families (Oei et al., 2018).

The pathogenesis of osteoporosis involves the interplay of multiple factors. Estrogen deficiency (Wang et al., 2023), elevated levels of pro-inflammatory cytokines (Yao et al., 2025), disruption of cellular redox homeostasis (Zhang et al., 2023), and genetic susceptibility (Zhu et al., 2021) all contribute to suppressed bone formation and enhanced bone resorption, ultimately leading to bone loss. Although several pharmacological agents have demonstrated efficacy in ameliorating osteoporosis, they are associated with adverse effects or are unsuitable for long-term use. For instance, intravenous bisphosphonates may carry a risk of nephrotoxicity, while oral bisphosphonates frequently cause upper gastrointestinal adverse reactions (Papapetrou, 2009). Denosumab, a monoclonal antibody targeting the receptor activator of nuclear factor-κB ligand (RANKL) pathway, has been associated with an increased risk of cellulitis (Reid, 2015). Therefore, identifying safer and more effective agents suitable for long-term administration to maintain the dynamic equilibrium of bone remodeling is of considerable clinical significance.

Active components derived from traditional Chinese medicine, as well as multi-herb formulations, demonstrate clinical value and distinct advantages in the management of osteoporosis. Naturally occurring polyphenols are being investigated as potential modulators of bone remodeling through antioxidant, anti-inflammatory, estrogen-related, and RANKL/OPG-associated pathways (Perrone et al., 2025). Gallic acid, for example, inhibited RANKL-induced osteoclastogenesis and attenuated OVX-induced bone loss in mice (Zhang et al., 2022). Multi-component traditional formulations are also being examined in this preclinical context. Yougui Pills were reported to reduce bone loss in OVX mice in association with suppression of the Th17/IL-17/NF-κB axis (Zhang et al., 2025). Puerarin, the major bioactive isoflavone isolated from the traditional Chinese herb *Pueraria lobata*, exerts a range of pharmacological activities, including modulation of bone metabolism (Xiao et al., 2020), anti-inflammatory effects (Zeng et al., 2022), and antioxidant properties (Ruan et al., 2025). Preclinical evidence suggests that puerarin can regulate bone metabolism, suppress bone resorption, and ameliorate inflammatory and oxidative stress states, thereby improving bone mineral density and bone microarchitecture. However, the specific mechanisms by which puerarin confers protection against osteoporosis remain incompletely understood. Although several meta-analyses of puerarin have been performed in rat models of osteoporosis (Yang et al., 2026), analyses of bone microarchitecture and mechanism-related indicators, including inflammation and oxidative stress, remain limited. In the present study, we systematically synthesized bone mineral density, bone microarchitecture, bone metabolism, inflammatory, and oxidative stress outcomes and summarized the putative bone-protective mechanisms of puerarin to inform future preclinical research.

## 2 Methods

This meta-analysis was designed and conducted in accordance with the Preferred Reporting Items for Systematic Reviews and Meta-Analyses (PRISMA) guidelines to ensure accuracy, transparency, and completeness. The study protocol was prospectively registered in the International Prospective Register of Systematic Reviews (PROSPERO; CRD420251236043).

### 2.1 Search strategy

Two investigators independently performed a systematic literature search for animal studies investigating the effects of puerarin on osteoporosis across seven databases: PubMed, Web of Science, Embase, CBM, CNKI, WanFang, and VIP. The search covered all records from database inception through February 1, 2026. The complete search strategies for each database are presented in **Supplementary Table 1**.

### 2.2 Inclusion and exclusion criteria

Inclusion criteria were defined according to the PICOS framework: (1) Population (P): animal models of osteoporosis; (2) Intervention (I): puerarin treatment; (3) Comparison (C): untreated controls or vehicle-treated controls without active pharmacological intervention; (4) Outcomes (O): primary outcomes included BMD, Tb.N, Tb.Sp, Tb.Th, BV/TV, and Conn.D; secondary outcomes included bone metabolism markers, inflammatory cytokines, and oxidative stress indices.(5) Study design (S): controlled *in vivo* animal intervention studies with at least one puerarin-monotherapy group and one untreated or vehicle-treated osteoporotic model control group, reporting quantitative outcome data for both groups.

Exclusion criteria were as follows: (1) duplicate publications; (2) reviews, network pharmacology studies, expert consensus statements, or technical reports; (3) observational studies, human studies, or *in vitro* experiments; (4) studies in which puerarin was not used as a monotherapy, combined with other interventions, or lacked a control group; (5) animal models with comorbid diseases; (6) full text unavailable; (7) studies in which outcome measures of interest were not reported or data were incomplete.

### 2.3 Data extraction

Two investigators independently screened the literature according to the eligibility criteria. After cross-verification of screening decisions, data were extracted using a standardized form. Any disagreements were resolved through discussion or consultation with a third investigator. Extracted data included: (1) study characteristics: first author name and publication year; (2) animal characteristics: species, strain, sex, body weight, and numbers of animals in experimental and control groups; (3) model establishment method; (4) pharmacological intervention: batch number documentation or purity required for commercial products, route of administration, dosage, and treatment duration; (5) outcome measures: BMD, Tb.N, Tb.Sp, Tb.Th, BV/TV, Conn.D, BGP, PINP, ALP, CTX-I, TRACP-5b, RANKL, OPG, IL-6, TNF-α, SOD, MDA, and GSH-Px. Data presented only in graphical form were extracted using WebPlotDigitizer 4.7 software. When studies reported the standard error of the mean (SEM), values were converted to standard deviation (SD) using the formula SD = SEM × $\sqrt{n}$. For studies reporting outcomes at multiple time points, only data from the final time point were used. When multiple doses were tested, data from the highest dose group were selected for the primary analyses.

### 2.4 Risk of bias assessment

Two investigators assessed the methodological quality of included studies using the SYRCLE risk-of-bias tool for animal studies. This instrument comprises 10 items addressing selection bias, performance bias, detection bias, attrition bias, reporting bias, and other sources of bias. Discrepancies in quality ratings were resolved through discussion with a third investigator. Quality assessment results were visualized using RevMan 5.3.

### 2.5 Statistical analysis

All statistical analyses were performed using STATA 14.0. Pooled effect sizes were expressed as standardized mean differences (SMD) with 95% confidence intervals (CIs), and a two-sided *p* < 0.05 was considered statistically significant. Heterogeneity across studies was assessed using the *I*² statistic. When *I*² > 50% or the Cochran’s Q test *p* < 0.1, indicating substantial heterogeneity, a random-effects model was applied; otherwise, a fixed-effects model was used. To explore potential sources of heterogeneity, sensitivity analyses (leave-one-out method) and subgroup analyses were conducted. Subgroup analyses were stratified by animal species (rat vs. mouse), route of puerarin administration (oral gavage vs. intraperitoneal injection vs. subcutaneous injection), dosage (≤ 50 mg/kg vs. > 50 mg/kg), and treatment duration (< 8 weeks vs. 8 to < 12 weeks vs. ≥ 12 weeks). Publication bias was assessed using funnel plots and Egger’s test, and the trim-and-fill method was applied when asymmetry was detected.

## 3 Results

### 3.1 Study selection

**Figure 1:**
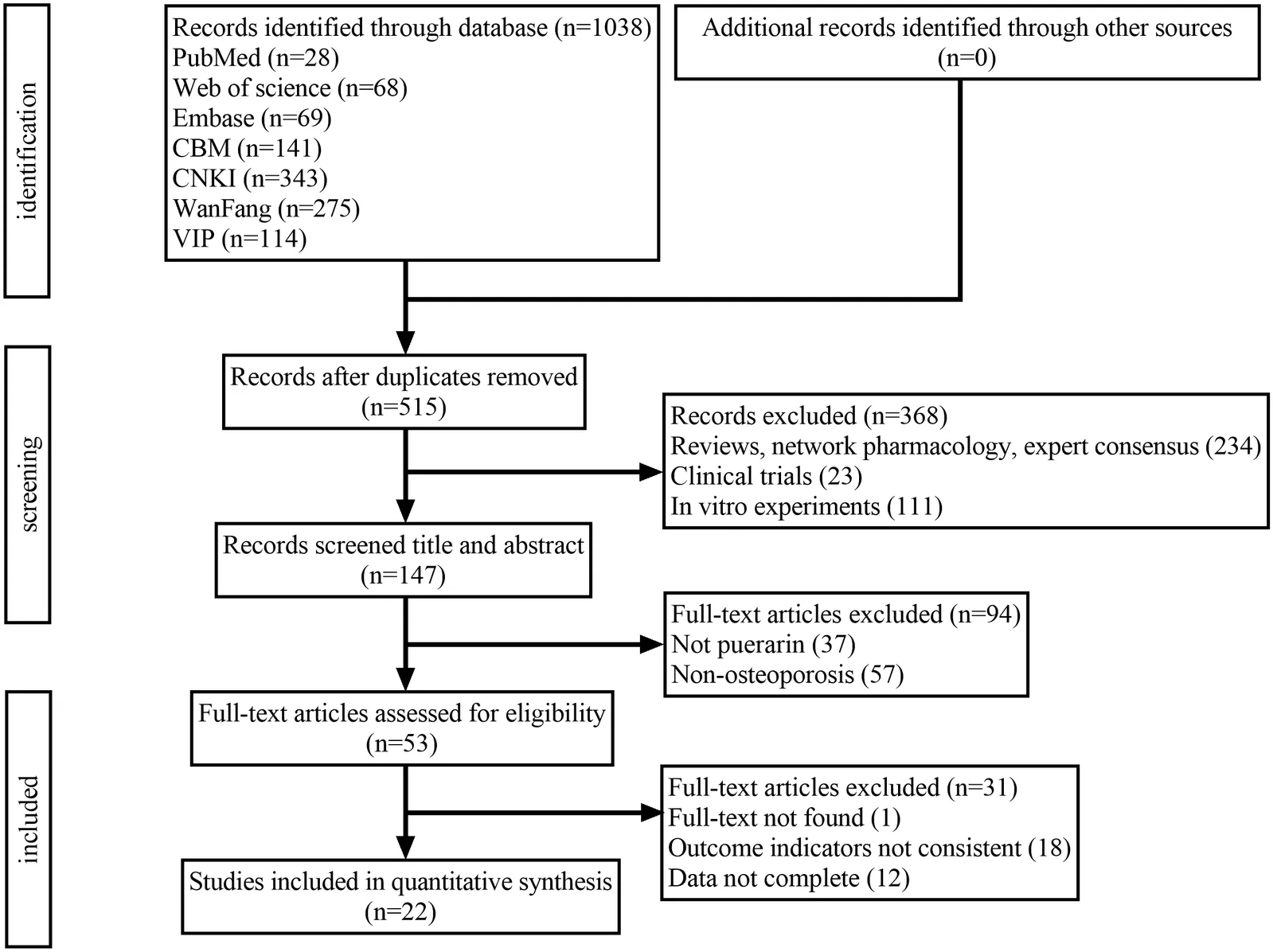
Flow chart of study selection.

A total of 1,038 records were retrieved from the seven databases. After removing duplicates, 515 records remained. Following title/abstract screening and full-text evaluation, 493 records were excluded, and 22 studies ultimately met the inclusion criteria for the meta-analysis (**Figure 1**).

### 3.2 Study characteristics

The 22 included studies encompassed 447 animals, with 224 in the treatment groups and 223 in the control groups. Regarding species and strain, 15 studies used Sprague–Dawley rats, 5 used Wistar rats, 1 used C57BL/6J mice, and 1 used C57BL/6 mice. All studies employed female animals, and osteoporosis was induced by bilateral ovariectomy (OVX) in every case. Body weight was reported in 20 studies. With respect to the route of puerarin administration, 10 studies employed subcutaneous injection, 3 used intraperitoneal injection, and 9 used oral gavage. Puerarin dosage ranged from 20 mg/kg to a maximum of 100 mg/kg. Treatment duration ranged from 4 weeks to 20 weeks. For primary outcomes, 21 studies reported BMD, 10 reported Tb.N, 8 reported Tb.Sp, 9 reported Tb.Th, 9 reported BV/TV, and 3 reported Conn.D. Twenty studies reported changes in bone metabolism markers, inflammatory cytokines, or oxidative stress indices. The characteristics of the included studies are summarized in **Table 1**. The batch numbers or purity on the puerarin was shown in **Supplementary Table 2**.

**Table 1:** Characteristics of the studies.

Author, year	Species (Sex, NE/NC)	Weight	Modeling method	Dose	Route	Duration	Outcomes
Chen and Hu, 2021	Wistar rats (female, 9/9)	230–260 g	ovariectomized	35 mg/kg/d	subcutaneous injection	6 weeks	BMD, ALP, BGP, TRACP-5b, IL-6, TNF-α, SOD, MDA, GSH-Px
Chen, 2024	SD rats (female, 10/10)	180–220 g	ovariectomized	100 mg/kg/d	gavage	8 weeks	BMD, BV/TV, Tb.Th, Tb.N, IL-6, TNF-α, SOD, GSH-Px
Feng, 2018	SD rats (female, 13/13)	226.80 ± 19.27 g	ovariectomized	100 mg/kg/d	subcutaneous injection	10 weeks	BMD, BV/TV, Tb.Th, Tb.N, Tb.Sp, Conn.D, OPG, RANKL, CTX-I, TRACP-5b, MDA, SOD, GSH-Px
Huang et al., 2011	SD rats (female, 5/5)	280 ± 20 g	ovariectomized	50 mg/kg/d	subcutaneous injection	20 weeks	BMD
Li and Zhang, 2009	Wistar rats (female, 9/8)	290.9 ± 1.5 g	ovariectomized	20 mg/kg/d	gavage	12 weeks	BMD, IL-6
Li et al., 2020	SD rats (female, 10/10)	NR	ovariectomized	100 mg/kg/d	gavage	14 weeks	BMD, Tb.Sp, BV/TV, Tb.N, Tb.Th, CTX-I, TRACP-5b
Li et al., 2021	Wistar rats (female, 10/10)	230–260 g	ovariectomized	35 mg/kg/d	subcutaneous injection	8 weeks	BMD, ALP, BGP, TRACP-5b, CTX-I, IL-6, TNF-α
Liang et al., 2012	SD rats (female, 9/9)	245–341 g	ovariectomized	20 mg/kg/d	gavage	12 weeks	BMD, OPG, ALP
Lv, 2006	SD rats (female, 10/10)	240–310 g	ovariectomized	20 mg/kg/d	gavage	3 months	BMD, Tb.Th, Tb.N, Tb.Sp, ALP, BGP
Lv and Fan, 2023	Wistar rats (female, 15/15)	180 ± 20 g	ovariectomized	35 mg/kg/d	subcutaneous injection	4 weeks	BMD, BV/TV, Tb.N, Tb.Th, Conn.D, Tb.Sp, CTX-I, PINP
Tang, 2005	SD rats (female, 12/12)	200 g	ovariectomized	100 mg/kg/d	subcutaneous injection	7 weeks	BMD, MDA
Wang and Lin, 2019	SD rats (female, 10/10)	225–258 g	ovariectomized	50 mg/kg/d	gavage	10 weeks	BMD, ALP, BGP, CTX-I, OPG, RANKL
Wen et al., 2025	Wistar rats (female, 10/10)	180–200 g	ovariectomized	100 mg/kg/d	gavage	3 months	BV/TV, Tb.N, Tb.Th, Tb.Sp, OPG
Xiao et al., 2020	C57BL/6J mice (female, 6/6)	NR	ovariectomized	100 mg/kg/d	intraperitoneal injection	6 weeks	BMD, BV/TV, Tb.N, Conn.D
Xu et al., 2021	C57BL/6 mice (female, 10/10)	24 ± 2 g	ovariectomized	100 mg/kg/d	gavage	8 weeks	BMD, BV/TV, Tb.Th, Tb.N, Tb.Sp, TRACP-5b
Yang et al., 2021	SD rats (female, 11/11)	230–260 g	ovariectomized	35 mg/kg/d	subcutaneous injection	6 weeks	BMD, TRACP-5b, RANKL, PINP, BGP, MDA, SOD
Yang and Guan, 2023	SD rats (female, 10/10)	200–257 g	ovariectomized	30 mg/kg/d	intraperitoneal injection	6 weeks	BMD, TRACP-5b, PINP, BGP, ALP, IL-6, TNF-α
Yu et al., 2019	SD rats (female, 5/5)	180–220 g	ovariectomized	40 mg/kg/d	subcutaneous injection	16 weeks	BMD, CTX-I
Yue et al., 2021	SD rats (female, 12/12)	320–350 g	ovariectomized	35 mg/kg/d	subcutaneous injection	6 weeks	BMD, RANKL, PINP, TRACP-5b, BGP
Zeng and Shi, 2018	SD rats (female, 10/10)	220–240 g	ovariectomized	100 mg/kg/d	gavage	3 months	BMD
Zhao et al., 2024	SD rats (female, 8/8)	200 ± 20 g	ovariectomized	80 mg/kg/d	subcutaneous injection	8 weeks	BMD, BV/TV, Tb.Th, Tb.N, Tb.Sp, PINP
Zhou et al., 2025	SD rats (female, 20/20)	240–260 g	ovariectomized	30 mg/kg/d	intraperitoneal injection	7 weeks	BMD, BV/TV, Tb.Th, Tb.N, Tb.Sp, ALP, PINP, IL-6

### 3.3 Quality assessment

**Figure 2:**
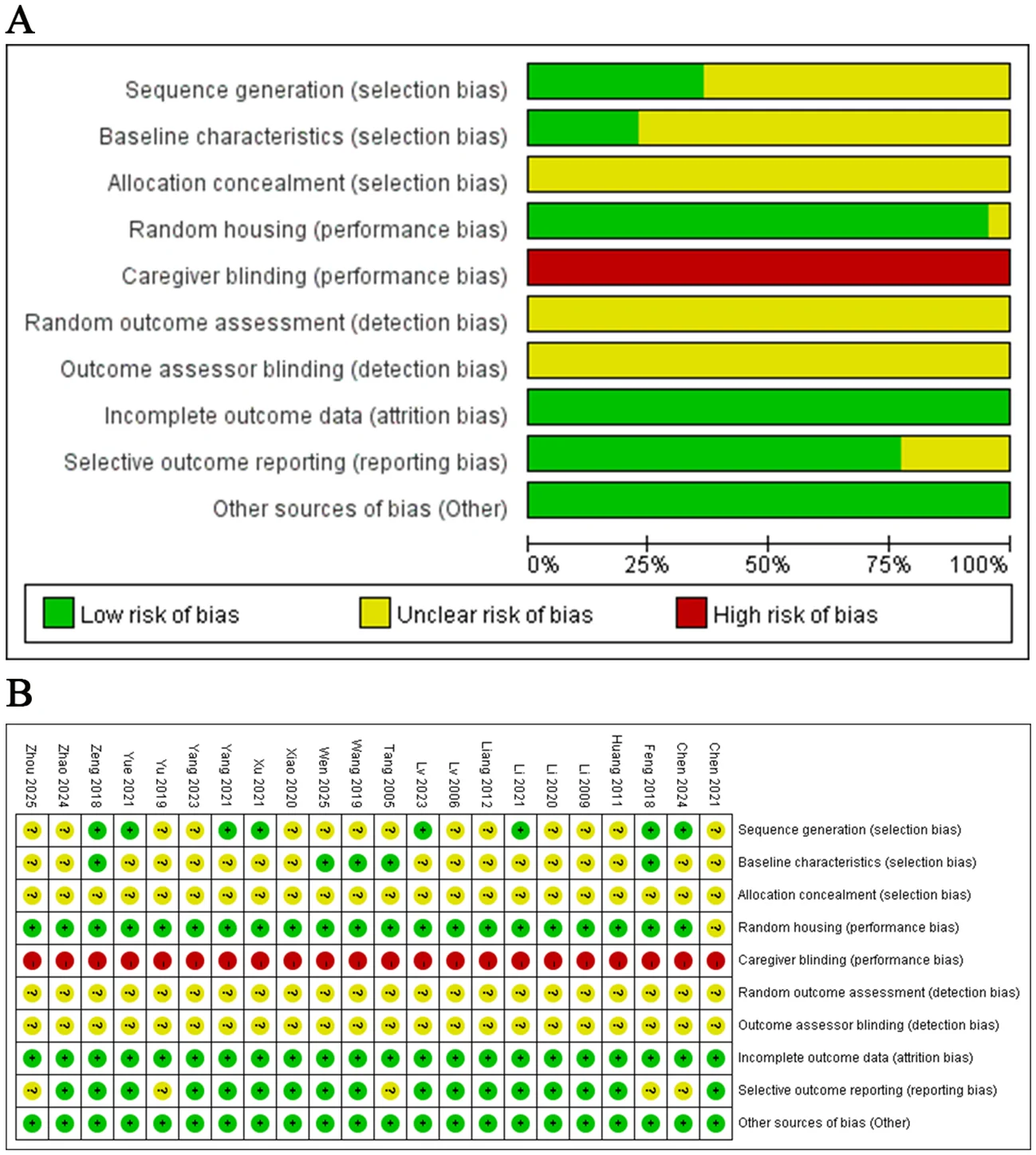
Deviation chart **(A)** and bias summary **(B)** incorporating study risks.

Methodological quality was assessed using the SYRCLE risk-of-bias tool, and the results are displayed using RevMan 5.3 (**Figure 2A**). Across the 22 studies, the domains of selective outcome reporting and other sources of bias were rated as low risk. Caregiver blinding was rated as high risk. Allocation concealment, random outcome assessment, and outcome assessor blinding were rated as unclear risk. Random housing was reported in 21 studies, sequence generation in 8 studies, baseline characteristics in 5 studies, and incomplete outcome data were noted in 6 studies (**Figure 2B**).

### 3.4 Primary outcomes

**Figure 3:**
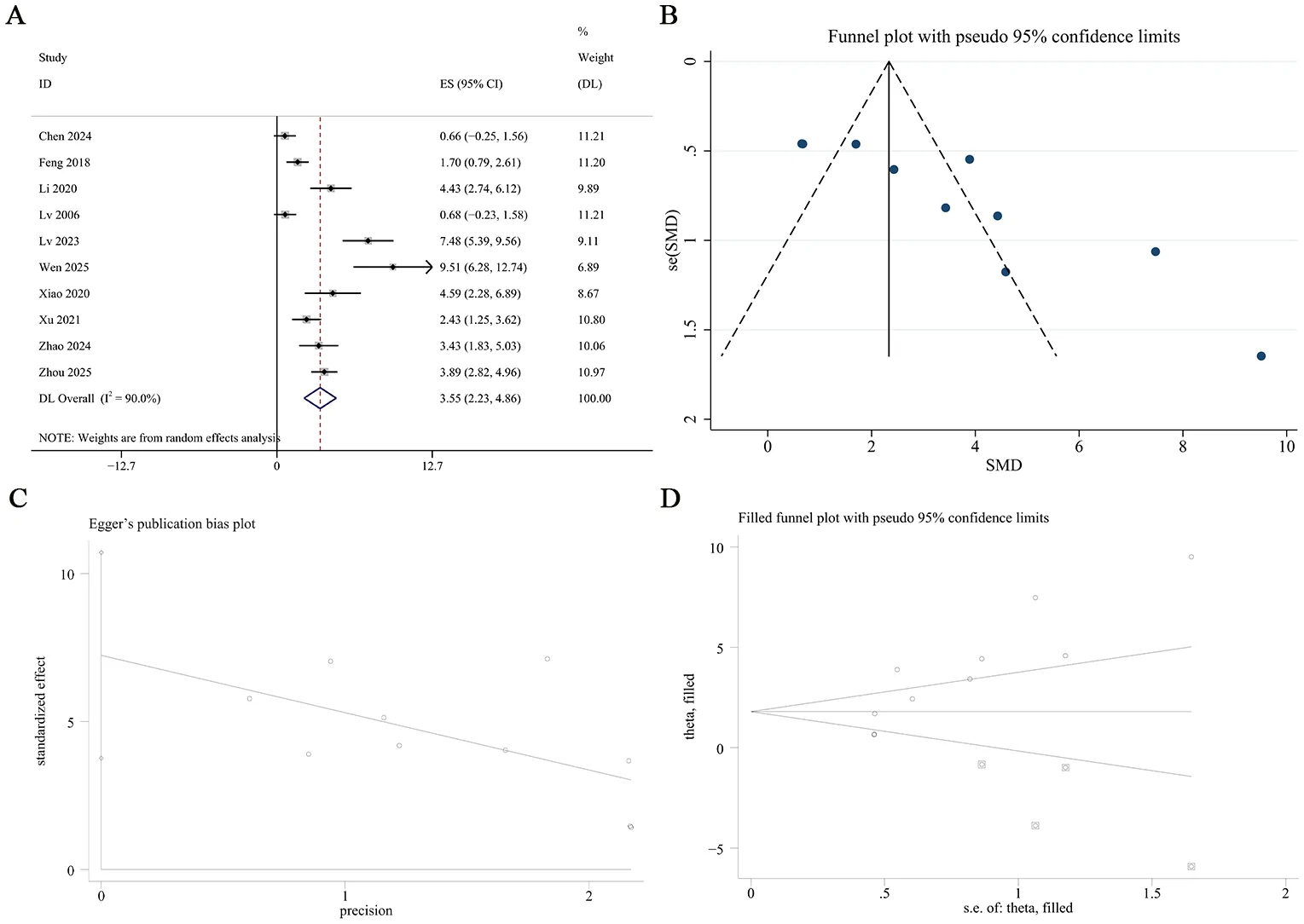
Effect of puerarin on BMD. **(A)** Forest plot. **(B)** Funnel plot. **(C)** Egger’s publication bias plot. **(D)** Filled funnel plot.

Bone mineral density (BMD). Twenty-one studies (Tang, 2005; Lv, 2006; Li and Zhang, 2009; Huang et al., 2011; Liang et al., 2012; Feng, 2018; Zeng and Shi, 2018; Wang and Lin, 2019; Yu et al., 2019; Li et al., 2020; Xiao et al., 2020; Li et al., 2021; Yue et al., 2021; Yang et al., 2021; Xu et al., 2021; Chen and Hu, 2021; Lv and Fan, 2023; Yang and Guan, 2023; Chen, 2024; Zhao et al., 2024; Zhou et al., 2025) reported BMD data. The meta-analysis showed that, compared with the model control group, puerarin significantly increased BMD [SMD: 2.62, 95% CI (2.02, 3.22), p < 0.001; I² = 79.6%, p < 0.001; **Figure 3A**]. Subgroup analysis revealed that heterogeneity was markedly decreased in the mouse subgroup (I² = 0.0%, p = 0.789) and the intraperitoneal injection subgroup (I² = 0.0%, p = 0.527) (**Supplementary Table 3**). A funnel plot was constructed (**Figure 3B**), and Egger’s test indicated statistically significant publication bias for BMD (p < 0.001; **Figure 3C**). The trim-and-fill method suggested that 7 hypothetical missing studies would be required to achieve funnel plot symmetry (**Figure 3D**; **Supplementary Table 4**).

**Figure 4:**
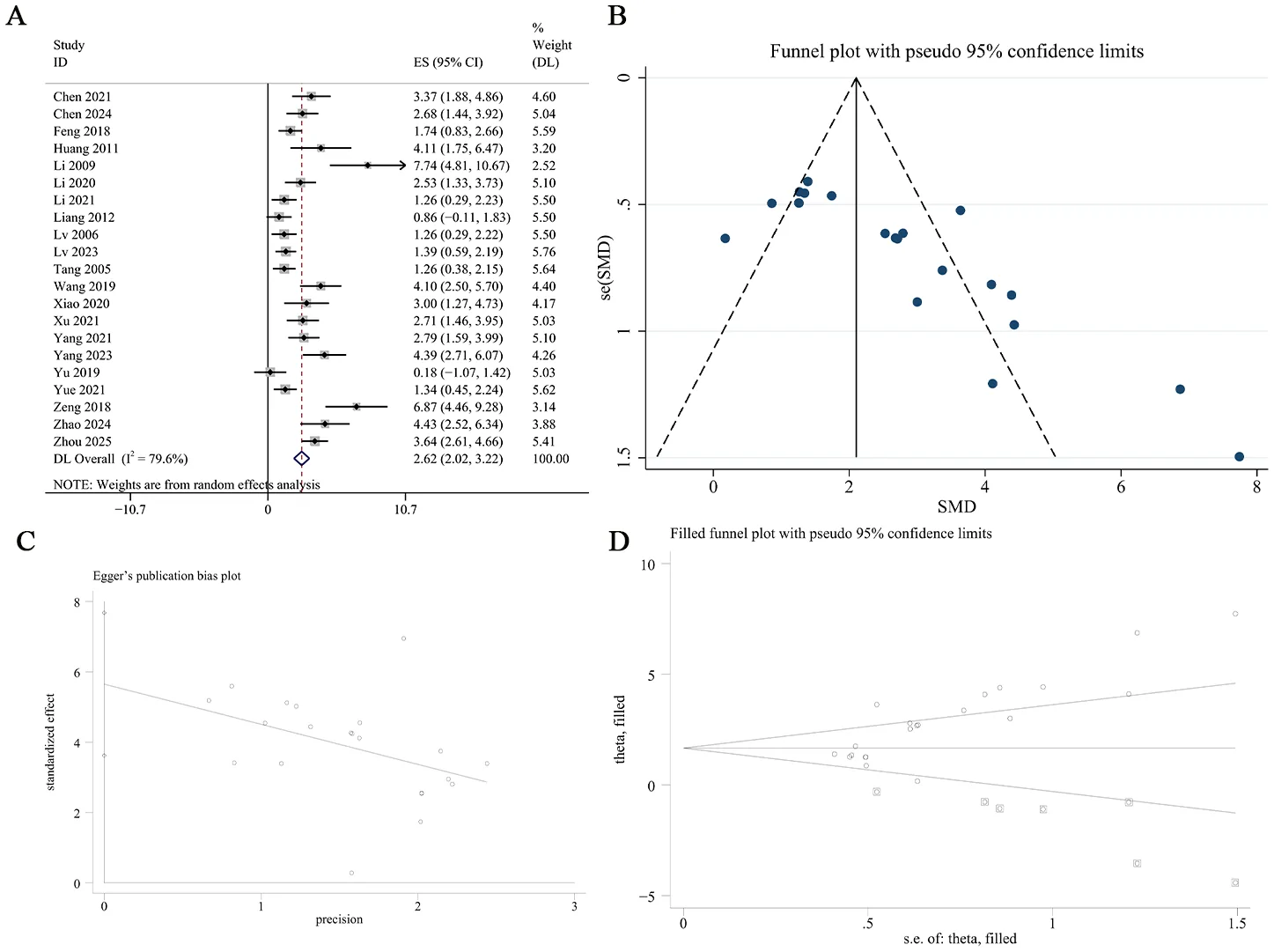
Effect of puerarin on Tb.N. **(A)** Forest plot. **(B)** Funnel plot. **(C)** Egger’s publication bias plot. **(D)** Filled funnel plot.

Trabecular number (Tb.N). Ten studies (Lv, 2006; Feng, 2018; Li et al., 2020; Xiao et al., 2020; Xu et al., 2021; Lv and Fan, 2023; Chen, 2024; Zhao et al., 2024; Wen et al., 2025; Zhou et al., 2025) reported Tb.N data. Puerarin significantly increased Tb.N compared with the model group [SMD: 3.55, 95% CI (2.23, 4.86), p < 0.001; I² = 90.0%, p < 0.001; **Figure 4A**]. Subgroup analyses did not reveal significant differences among subgroups (**Supplementary Table 3**). Egger’s test indicated significant publication bias (p = 0.001; **Figures 4B, C**), and the trim-and-fill analysis suggested 4 missing studies to restore funnel symmetry (**Figure 4D**; **Supplementary Table 4**).

**Figure 5:**
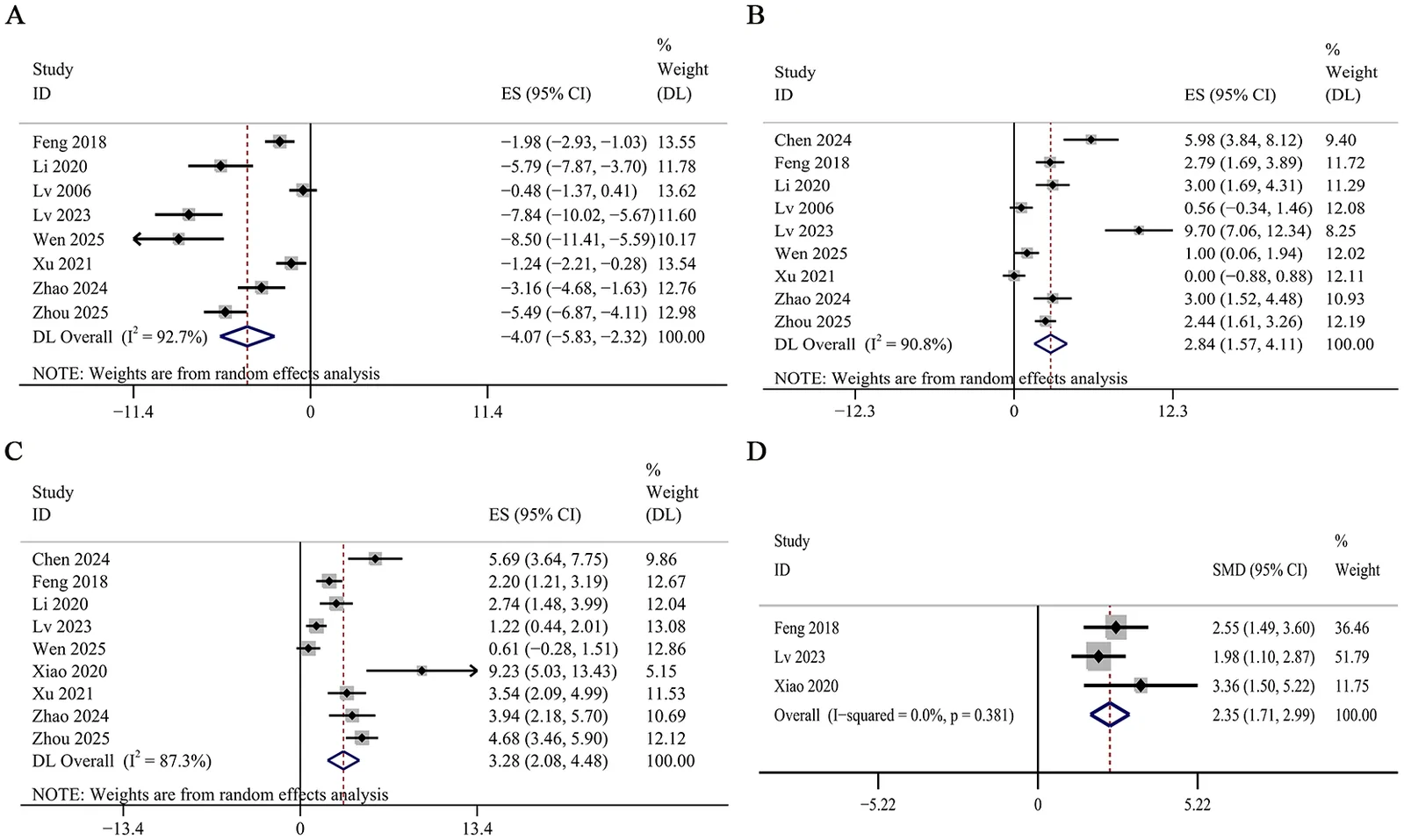
Forest plot of the effect of puerarin on **(A)** Tb.Sp, **(B)** Tb.Th, **(C)** BV/TV, **(D)** Conn.D.

Trabecular separation (Tb.Sp). Eight studies (Lv, 2006; Feng, 2018; Li et al., 2020; Xu et al., 2021; Lv and Fan, 2023; Zhao et al., 2024; Wen et al., 2025; Zhou et al., 2025) evaluated Tb.Sp. Puerarin significantly reduced Tb.Sp [SMD: −4.07, 95% CI (−5.83, −2.32), p < 0.001; I² = 92.7%, p < 0.001; **Figure 5A**].

Trabecular thickness (Tb.Th). Nine studies (Lv, 2006; Feng, 2018; Li et al., 2020; Xu et al., 2021; Lv and Fan, 2023; Chen, 2024; Zhao et al., 2024; Wen et al., 2025; Zhou et al., 2025) assessed Tb.Th. Puerarin significantly increased Tb.Th [SMD: 2.84, 95% CI (1.57, 4.11), p < 0.001; I² = 90.8%, p < 0.001; **Figure 5B**].

Bone volume fraction (BV/TV). Nine studies (Feng, 2018; Li et al., 2020; Xiao et al., 2020; Xu et al., 2021; Lv and Fan, 2023; Chen, 2024; Zhao et al., 2024; Wen et al., 2025; Zhou et al., 2025) reported BV/TV. Puerarin significantly increased BV/TV [SMD: 3.28, 95% CI (2.08, 4.48), p < 0.001; I² = 87.3%, p < 0.001; **Figure 5C**].

Connectivity density (Conn.D). Three studies (Feng, 2018; Xiao et al., 2020; Lv and Fan, 2023) evaluated Conn.D. Puerarin significantly increased Conn.D [SMD: 2.35, 95% CI (1.71, 2.99), p < 0.001; I² = 0.0%, p = 0.381; **Figure 5D**].

### 3.5 Secondary outcomes

#### 3.5.1 Bone formation markers

**Figure 6:**
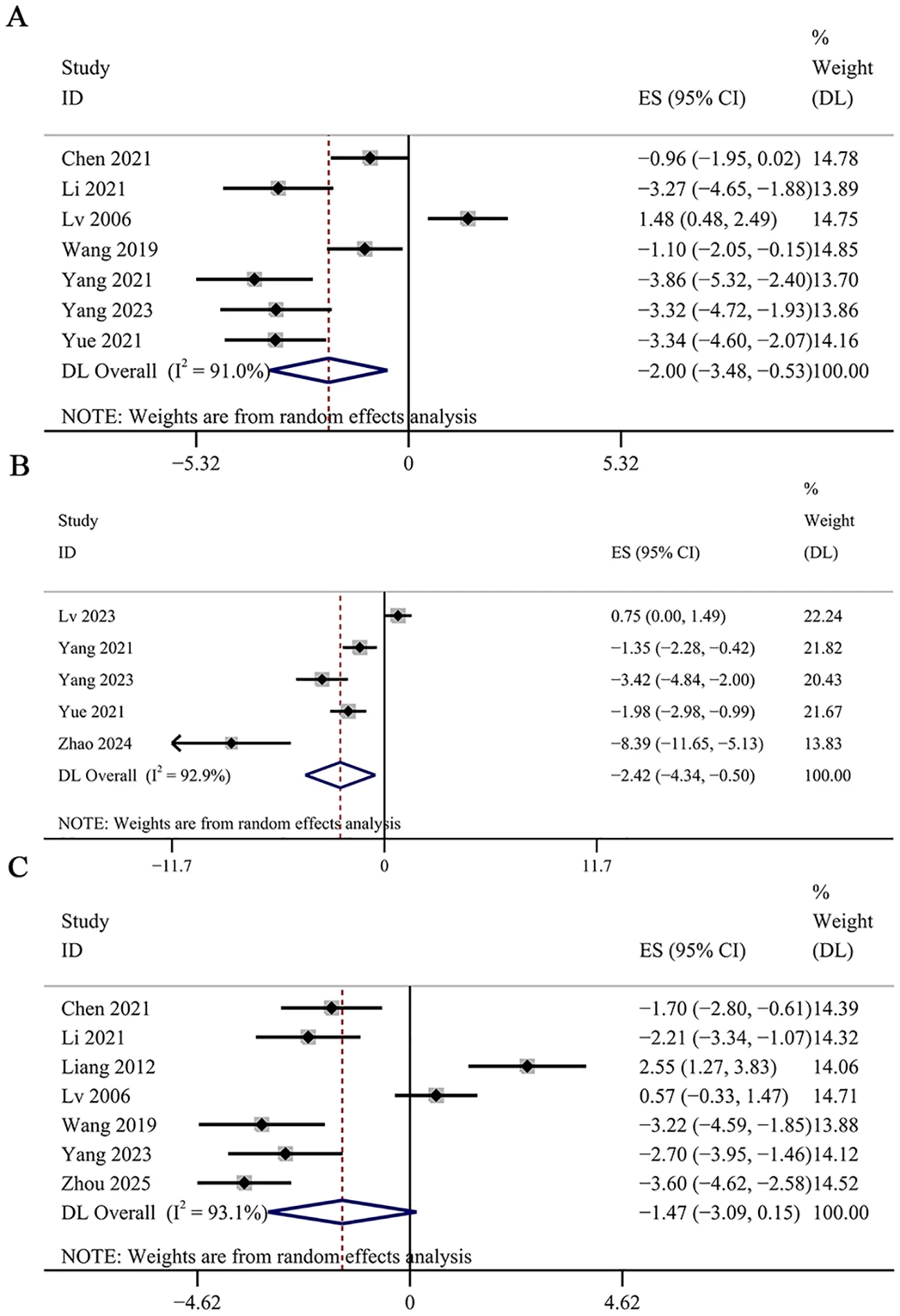
Forest plot of the effect of puerarin on **(A)** BGP, **(B)** PINP, **(C)** ALP.

Seven studies (Lv, 2006; Wang and Lin, 2019; Chen and Hu, 2021; Li et al., 2021; Yang et al., 2021; Yue et al., 2021; Yang and Guan, 2023) examined BGP and ALP. Six studies (Yang et al., 2021; Yue et al., 2021; Lv and Fan, 2023; Yang and Guan, 2023; Zhao et al., 2024; Zhou et al., 2025) assessed PINP, and five were retained in the pooled analysis after sensitivity analysis. Compared with the model group, puerarin significantly reduced BGP [SMD: −2.00, 95% CI (−3.48, −0.53), p = 0.008; I² = 91.0%, p < 0.001; **Figure 6A**] and PINP [SMD: −2.42, 95% CI (−4.34, −0.50), p = 0.013; I² = 92.9%, p < 0.001; **Figure 6B**]. The reduction in ALP did not reach statistical significance [SMD: −1.47, 95% CI (−3.09, 0.15), p = 0.075; I² = 93.1%, p < 0.001; **Figure 6C**].

#### 3.5.2 Bone resorption markers

**Figure 7:**
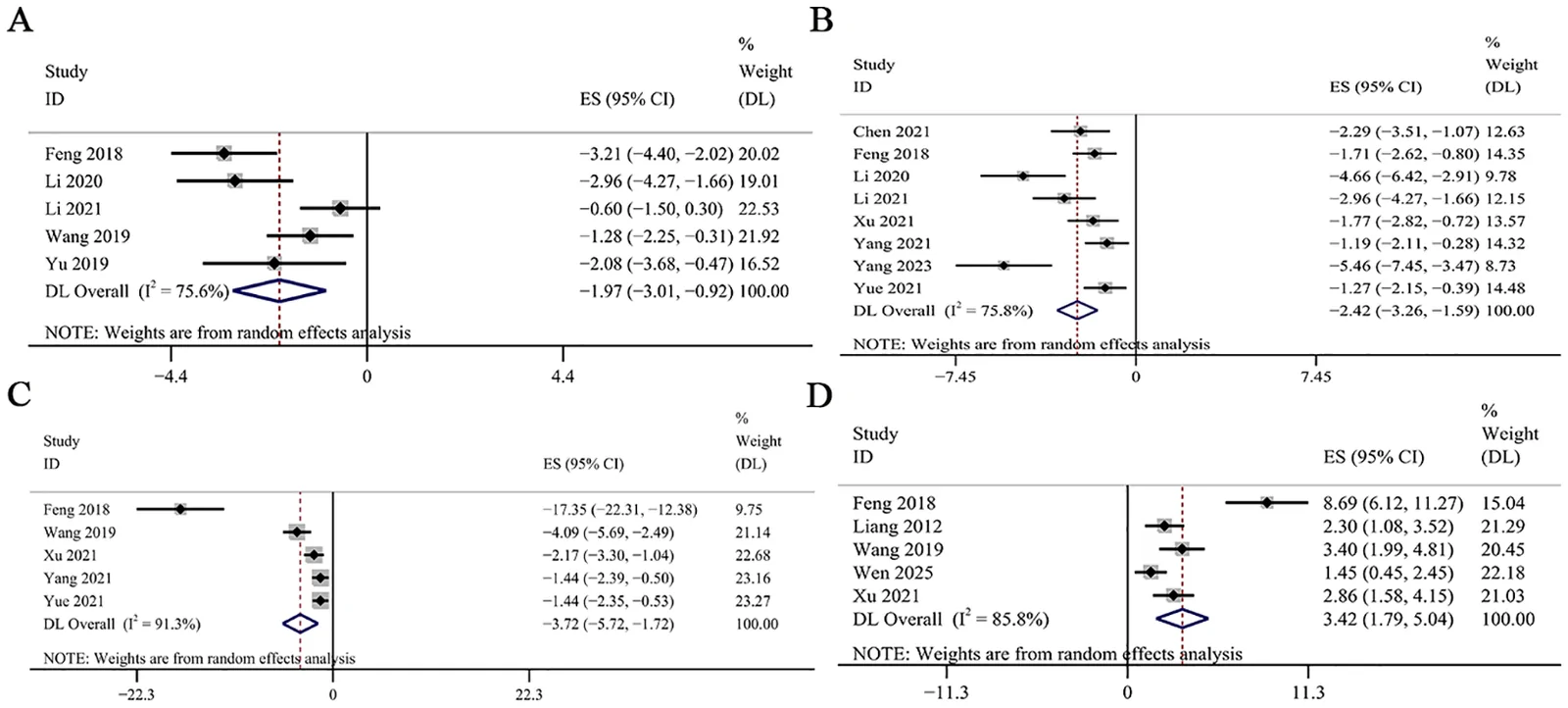
Forest plot of the effect of puerarin on **(A)** CTX-I, **(B)** TRACP-5b, **(C)** RANKL, **(D)** OPG.

Six studies (Feng, 2018; Wang and Lin, 2019; Yu et al., 2019; Li et al., 2020, 2021) reported CTX-I levels, and five were retained in the pooled analysis after sensitivity analysis; eight studies (Feng, 2018; Li et al., 2020; Chen and Hu, 2021; Li et al., 2021; Xu et al., 2021; Yang et al., 2021; Yue et al., 2021; Yang and Guan, 2023) reported TRACP-5b levels, and five evaluated RANKL (Feng, 2018; Wang and Lin, 2019; Xu et al., 2021; Yang et al., 2021; Yue et al., 2021) and OPG levels (Feng, 2018; Wang and Lin, 2019; Xu et al., 2021; Yang et al., 2021; Yue et al., 2021).Compared with the model group, puerarin significantly decreased CTX-I [SMD: −1.97, 95% CI (−3.01, −0.92), p < 0.001; I² = 75.6%, p < 0.001; **Figure 7A**], TRACP-5b [SMD: −2.42, 95% CI (−3.26, −1.59), p < 0.001; I² = 75.8%, p < 0.001; **Figure 7B**], and RANKL [SMD: −3.72, 95% CI (−5.72, −1.72), p < 0.001; I² = 91.3%, p < 0.001; **Figure 7C**], while significantly increasing OPG [SMD: 3.42, 95% CI (1.79, 5.04), p < 0.001; I² = 85.8%, p < 0.001; **Figure 7D**].

#### 3.5.3 Inflammatory markers

**Figure 8:**
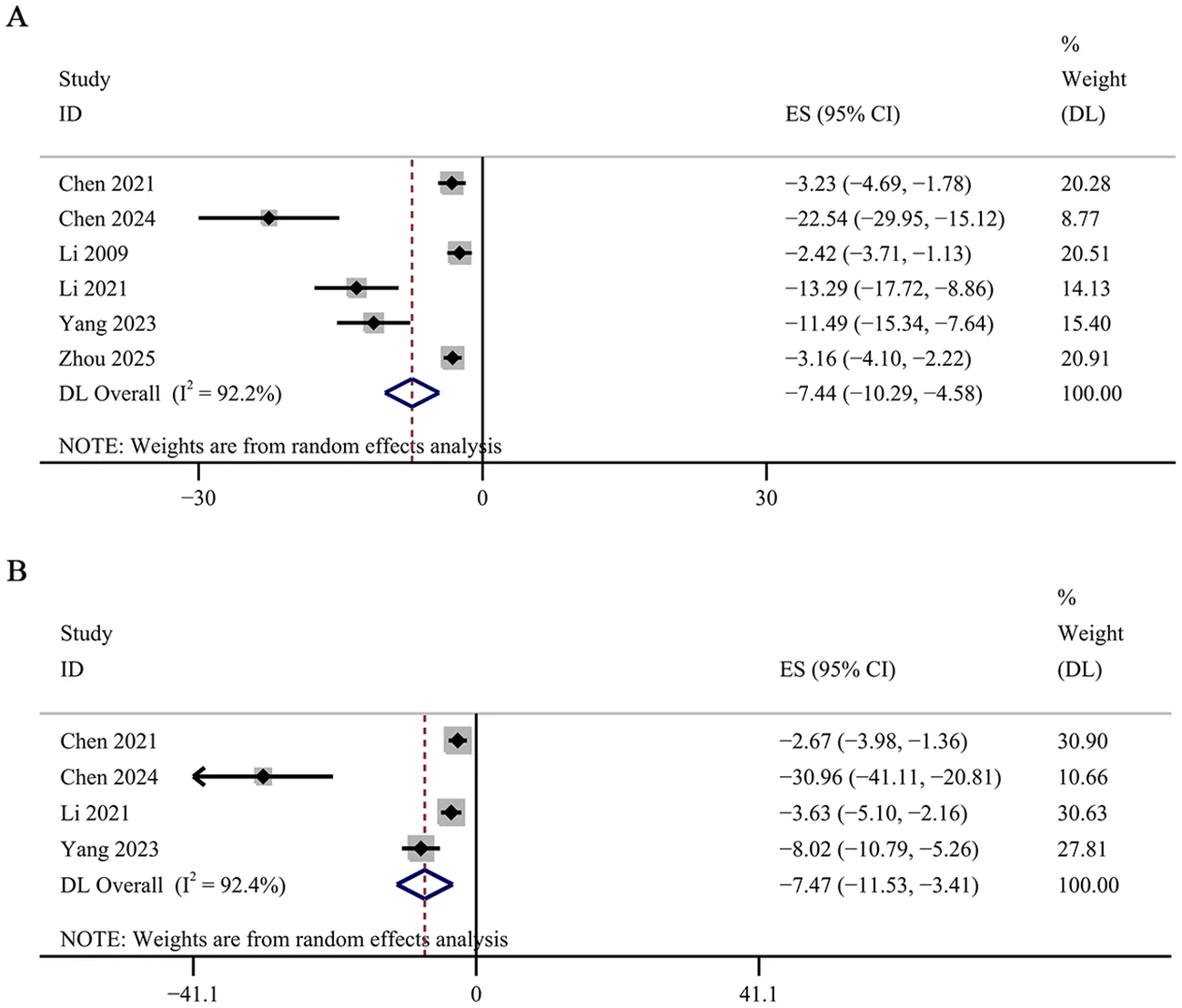
Forest plot of the effect of puerarin on **(A)** IL-6, **(B)** TNF-α.

Six studies (Li and Zhang, 2009; Chen and Hu, 2021; Li et al., 2021; Yang and Guan, 2023; Chen, 2024; Zhou et al., 2025) assessed IL-6 and four Chen and Hu, 2021; Li et al., 2021; Yang and Guan, 2023; Chen, 2024) evaluated TNF-α. Puerarin significantly reduced IL-6 [SMD: −7.44, 95% CI (−10.29, −4.58), p < 0.001; I² = 92.2%, p < 0.001; **Figure 8A**] and TNF-α [SMD: −7.47, 95% CI (−11.53, −3.41), p < 0.001; I² = 92.4%, p < 0.001; **Figure 8B**] compared with the model group.

#### 3.5.4 Oxidative stress markers

**Figure 9:**
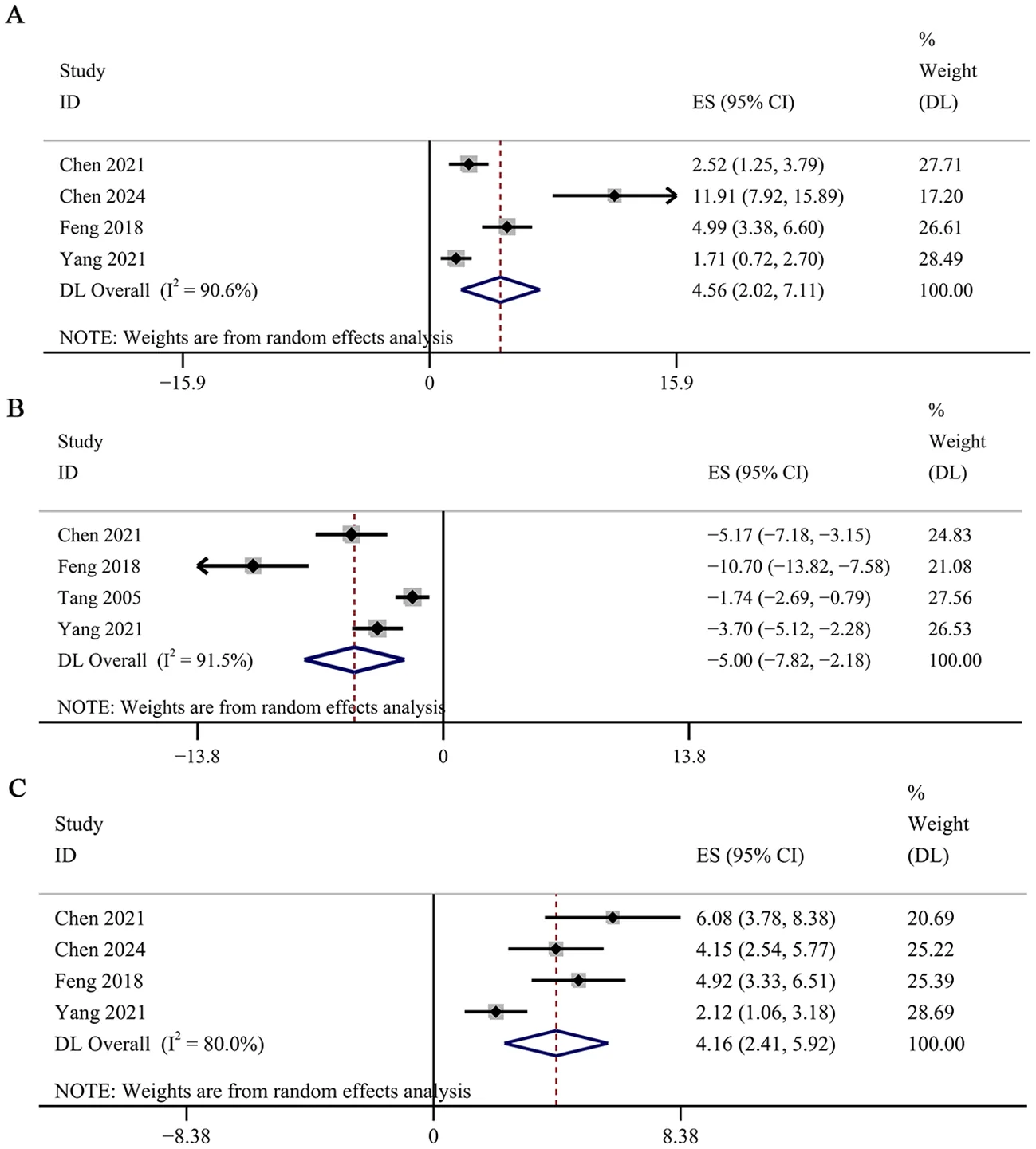
Forest plot of the effect of puerarin on **(A)** SOD, **(B)** MDA, **(C)** GSH-Px.

Four studies examined SOD (Feng, 2018; Chen and Hu, 2021; Yang et al., 2021; Chen, 2024), MDA (Feng, 2018; Chen and Hu, 2021; Yang et al., 2021; Chen, 2024), and GSH-Px (Feng, 2018; Chen and Hu, 2021; Yang et al., 2021; Chen, 2024). Puerarin significantly increased SOD [SMD: 4.56, 95% CI (2.02, 7.11), p < 0.001; I² = 90.6%, p < 0.001; **Figure 9A**] and GSH-Px [SMD: 4.16, 95% CI (2.41, 5.92), p < 0.001; I² = 80.0%, p < 0.001; **Figure 9C**], and significantly decreased MDA [SMD: −5.00, 95% CI (−7.82, −2.18), p < 0.001; I² = 91.5%, p < 0.001; **Figure 9B**].

### 3.6 Sensitivity analysis

Sensitivity analyses were performed for the primary outcomes (BMD, Tb.N, Tb.Sp, Tb.Th, BV/TV, and Conn.D) using a leave-one-out approach. The pooled effect estimates remained stable after sequential exclusion of individual studies, indicating that no single study disproportionately influenced the overall results (**Supplementary Figure 1**).

## 4 Discussion

### 4.1 Summary of key findings

This systematic review integrated data from 22 preclinical studies, in which osteoporosis models were all established via bilateral ovariectomy. The meta-analysis indicated that puerarin has significant bone-protective effects in experimental models of postmenopausal osteoporosis, evidenced by improvements in BMD and bone microarchitecture parameters (Tb.N, Tb.Th, BV/TV, and Conn.D) along with reductions in Tb.Sp. Puerarin also decreased levels of bone resorption markers (CTX-I, TRACP-5b, and RANKL) and increased OPG, suggesting that its bone-protective effect is predominantly mediated through the suppression of osteoclastogenesis and bone resorption.

Notably, the bone formation markers BGP and PINP were also significantly decreased, and ALP showed no significant change, indicating that the *in vivo* evidence does not support a straightforward bone-formation-promoting effect of puerarin. A more plausible interpretation is that, in the context of OVX-induced high bone turnover, puerarin simultaneously reduces both bone resorption and certain bone formation markers, with the dominant effect being the suppression of bone resorption, thereby rectifying the abnormally elevated bone turnover state. Furthermore, puerarin reduced IL-6, TNF-α, and MDA levels while increasing SOD and GSH-Px, suggesting that anti-inflammatory and antioxidant mechanisms may jointly contribute to its bone-protective effects, although this inference requires further empirical validation. Beyond BMD, our meta-analysis systematically incorporated multidimensional outcomes, including bone microarchitecture, bone metabolism, inflammation, and oxidative stress indices. The included animal studies exhibited considerable diversity in species selection, route of administration, dosage, and treatment duration. With respect to methodological quality, we recommend that future studies ensure adequate reporting of sequence generation, allocation concealment, and baseline characteristics, provide complete outcome data, and implement measures to minimize various sources of bias.

Considerable heterogeneity was observed in the primary outcomes. Subgroup analyses indicated that animal species and route of administration were potential sources of heterogeneity for BMD. The sources of heterogeneity cannot be completely elucidated, given the small sample sizes in each subgroup and the presence of confounders such as dose and route of administration. Although puerarin used across the included studies met pharmacopoeia quality standards, few studies characterized the chemical composition of their puerarin preparations, which may constitute an important source of heterogeneity. This underscores the need for strengthened standardization and transparent reporting of puerarin in future animal and clinical investigations. Egger’s test detected publication bias for BMD and Tb.N. Despite the use of a trim-and-fill adjustment, the preferential reporting of positive outcomes in the primary studies could still have led to an overestimation of the therapeutic effects of puerarin. We recommend that future studies adhere to rigorous reporting standards for randomization sequence, allocation concealment, baseline characteristics, and test substance characterization.

This study provides a comprehensive analysis of the effects of puerarin in ovariectomized animal models of osteoporosis and identifies priorities for further preclinical investigation. Clinical relevance remains to be established. It should be acknowledged that the included studies were limited to a single method of osteoporosis modeling, involving only ovariectomy. Therefore, the effects of puerarin on other forms of osteoporosis still require further exploration. Regarding mechanistic research, there is currently no comprehensive assessment of the precise molecular pathways underlying the action of puerarin on osteoporosis. It is necessary to conduct more rigorous preclinical investigations to clarify these mechanisms and strengthen the basis for translational medicine.

### 4.2 Putative protective mechanisms of puerarin

#### 4.2.1 Inhibition of osteoclastogenesis and bone resorption

Bone homeostasis relies on the dynamic equilibrium between osteoblast-mediated bone formation and osteoclast-mediated bone resorption. When this balance is disrupted and bone resorption persistently exceeds bone formation, net bone loss ensues (Kim et al., 2020). Factors such as hormonal changes, metabolic disorders, and glucocorticoid exposure can increase osteoclast number or activity, thereby driving the initiation and progression of osteoporosis (Eastell et al., 2016). In the present analysis, puerarin significantly reduced levels of the bone resorption markers CTX-I, TRACP-5b, and RANKL while upregulating OPG, suggesting that its bone-protective effects are predominantly attributable to suppression of osteoclastogenesis and bone resorption. The potential molecular mechanisms are outlined below.

Osteoclasts derive from monocyte/macrophage precursors of hematopoietic stem cell lineage, and the RANKL/RANK/OPG axis constitutes the core signaling pathway governing their differentiation and maturation. Binding of RANKL to RANK on the surface of osteoclast precursor cells recruits the intracellular adaptor protein TRAF6, which primarily activates downstream reactive oxygen species (ROS) production via NADPH oxidase (NOX) and the MAPK/NF-κB signaling cascade, thereby inducing the master transcription factor NFATc1 and initiating the transcription of osteoclast effector genes, including TRAP, cathepsin K (CTSK), and MMP-9 (Park et al., 2017). Upon functional activation, mature osteoclasts form the F-actin ring—a specialized cytoskeletal structure for bone resorption—through integrin αvβ3-mediated adhesive signaling and activation of the Pyk2/Src/Cbl signaling axis (Takito et al., 2018). Multiple studies have demonstrated that puerarin reduces the number of TRAP-positive osteoclasts in bone tissue, downregulates RANKL expression, upregulates OPG, and decreases the RANKL/OPG ratio (Liang et al., 2019; Xu et al., 2021). Xiao et al. (2020) confirmed in OVX mice *in vivo* and in RANKL-induced RAW264.7 cells and bone marrow-derived macrophages (BMMs) *in vitro* that puerarin downregulates TRAF6 and NOX1 protein expression, reduces intracellular ROS, suppresses osteoclast differentiation, and inhibits hydroxyapatite matrix resorption. From the perspective of osteoclast functional activation, Qiu et al. (2022) demonstrated that puerarin significantly inhibits F-actin ring formation and bone resorption activity without markedly affecting RANKL-induced osteoclast differentiation or apoptosis; mechanistically, this may be associated with decreased integrin β3 expression and reduced phosphorylation of Pyk2, Src, and Cbl, and puerarin treatment also improved trabecular microarchitecture and biomechanical properties in OVX rats. In addition, autophagy participates in the energy metabolism and differentiation program of osteoclast precursors, and the autophagy-related protein BECN1 has been shown to play a role in RANKL-induced osteoclastogenesis (Montaseri et al., 2020). Preliminary evidence from one study (Zhang et al., 2019) indicates that puerarin suppresses the proliferation and differentiation of osteoclast precursors by inhibiting both RANKL-dependent and RANKL-independent autophagic responses; notably, BECN1 overexpression significantly reverses the inhibitory effect of puerarin in the presence of RANKL. Taken together, the inhibitory effect of puerarin on osteoclasts likely spans multiple regulatory nodes, including osteoclast differentiation signaling, functional activation of bone resorption, and autophagy-mediated metabolic support.

#### 4.2.2 Modulation of osteoblast differentiation and bone formation

Osteoblasts originate from bone marrow mesenchymal stem cells (BMSCs) and are responsible for synthesizing type I collagen and other bone matrix proteins and regulating mineralization; their proliferation, differentiation, and survival directly determine the rate of bone formation (Capulli et al., 2014; Ponzetti and Rucci, 2021). Impaired osteoblast function or an imbalance between bone formation and bone resorption exacerbates net bone loss and contributes to the development of osteoporosis (Compston et al., 2019). It should be noted that in the present meta-analysis, the bone formation markers BGP and PINP were significantly decreased and ALP showed no significant change; therefore, the evidence supporting a bone-formation-promoting role of puerarin remains inconclusive. Nevertheless, multiple *in vitro* studies have suggested that puerarin possesses pro-osteogenic potential at the cellular level, and its mechanism of action must be interpreted in the context of the prevailing bone turnover state.

Osteogenic differentiation is regulated by multiple signaling pathways. The Wnt/β-catenin pathway serves as the core regulator of osteoblast maturation and matrix mineralization: upon nuclear translocation, β-catenin partners with TCF/LEF transcription factors to initiate osteogenic gene transcription (Baron and Kneissel, 2013). Within the TGF-β superfamily, BMP-2 signals through the Smad1/5/8 pathway to induce expression of the key osteogenic transcription factors Runx2 and Osterix (Wu et al., 2016, 2024). The NO/cGMP pathway also modulates osteoblast proliferation and differentiation (Kalyanaraman et al., 2018). *In vitro* and individual *in vivo* studies have demonstrated that puerarin modulates each of these pathways to varying degrees. Wang et al. (2012) reported that puerarin stimulates ALP activity, type I collagen secretion, and mineralized nodule formation in primary rat osteoblasts, and upregulates phosphorylated p38 MAPK and β-catenin protein in a time-dependent manner. Liang et al. (2012) found that puerarin upregulates BMP-2 expression and increases OPG in the bone tissue of OVX rats. Lv et al. (2015) confirmed in human BMSCs that puerarin promotes cell proliferation, ALP activity, and calcium deposition via the NO/cGMP pathway, and upregulates Runx2 and Osterix mRNA expression.

The pro-osteogenic evidence cited above derives largely from *in vitro* or individual studies; however, our pooled analysis found that *in vivo* bone formation markers were not elevated in animal models, and BGP and PINP were instead significantly decreased. This suggests that, under OVX-induced high bone turnover conditions, puerarin acts primarily by suppressing bone resorption, with bone formation markers declining in parallel—reflecting a correction of the abnormally high bone turnover state rather than a net increase in bone formation. Whether the pro-osteogenic effects observable *in vitro* can translate into a net gain in bone formation *in vivo* remains uncertain. Additionally, some of the *in vitro* evidence was obtained from the MG-63 osteosarcoma cell line, whose biological behavior differs from that of primary osteoblasts, and the inherent disparities between *in vitro* systems and the *in vivo* microenvironment further complicate interpretation. Consequently, additional high-quality basic research is needed to resolve this issue. Future investigations should validate the reproducibility of the aforementioned pathways in primary osteoblast or BMSC systems and incorporate dynamic bone histomorphometric parameters to assess the genuine effect of puerarin on bone formation *in vivo*.

#### 4.2.3 Anti-inflammatory effects

Chronic inflammation represents a shared pathological underpinning of multiple types of osteoporosis. Activated immune cells release pro-inflammatory cytokines such as TNF-α, IL-1β, and IL-6 (Ponzetti and Rucci, 2019), which upregulate RANKL expression on osteoblasts and stromal cells, downregulate OPG, promote the recruitment and differentiation of osteoclast precursors, and impair bone formation by suppressing Wnt signaling in osteoblasts and inducing osteoblast apoptosis (Weitzmann and Ofotokun, 2016). Our results demonstrated that puerarin significantly reduced IL-6 and TNF-α levels, a finding concordant with the observed downregulation of RANKL, upregulation of OPG, and improvements in bone microarchitecture, thus suggesting that anti-inflammatory effects may contribute to its bone-protective properties.

TNF-α directly induces osteoclast precursor differentiation through NF-κB pathway activation, an effect that is synergistically amplified in the presence of RANKL but can also independently drive partial osteoclastogenesis in its absence (Lam et al., 2000). IL-6, acting through membrane-bound or soluble IL-6 receptors (sIL-6R), activates the JAK/STAT3 pathway, which both stimulates RANKL expression in stromal cells and enhances the responsiveness of osteoclast precursors (McGregor et al., 2019). IL-1β promotes osteoclast activity and survival via the MAPK pathway (Lee et al., 2018). Multiple animal studies have reported that puerarin reduces serum or bone tissue levels of TNF-α and IL-6, accompanied by RANKL downregulation and OPG upregulation (Chen and Hu, 2021; Li et al., 2021; Yang and Guan, 2023; Chen, 2024). Tang et al. (2020), using a titanium particle-induced rat osteolysis model, demonstrated that puerarin reduces the number of IL-6- and TNF-α-positive cells in osteolytic lesions, downregulates NFATc1 and MMP-9, and improves trabecular microarchitecture; *in vitro*, puerarin also inhibited RANKL-induced NF-κB p65 phosphorylation and IκBα degradation in RAW264.7 cells and BMMs. Thus, the inhibition of inflammatory signaling by puerarin converges with its suppression of osteoclastogenesis at the NF-κB/NFATc1 pathway nexus, and the interconnectedness of these two mechanisms constitutes an important component of its comprehensive bone-protective network. However, given the relatively small number of studies reporting inflammatory outcomes and the high heterogeneity among them, the anti-inflammatory evidence remains exploratory and requires further validation.

#### 4.2.4 Anti-oxidative stress effects

Oxidative stress is an independent risk factor for the initiation and progression of osteoporosis. Under various pathological conditions, including aging and hormonal changes, excessive accumulation of ROS and attenuation of antioxidant defenses within the bone marrow microenvironment promote osteoclast precursor differentiation while inducing apoptosis of osteoblasts and osteocytes (Domazetovic et al., 2017). Under normal metabolic conditions, ROS are continuously generated and scavenged by antioxidant enzymes such as superoxide dismutase (SOD) and glutathione peroxidase (GSH-Px). When ROS production exceeds the scavenging capacity, oxidative stress ensues, resulting in the accumulation of lipid peroxidation products such as malondialdehyde (MDA), which directly damage cellular structures and functions (Marcucci et al., 2023). Among antioxidant defense mechanisms, the transcription factor Nrf2 serves as a central regulator, initiating the transcription of downstream genes including HO-1 and SOD upon oxidative stimulation (Baird and Yamamoto, 2020). The FoxO family of transcription factors constitutes an additional redox regulatory axis, upregulating antioxidant enzymes such as catalase and suppressing ROS accumulation to maintain bone homeostasis (Bartell et al., 2014).

In the present meta-analysis, puerarin significantly increased SOD and GSH-Px levels and decreased MDA levels, consistent with the observed improvements in BMD and bone microarchitecture. Multiple animal studies have similarly reported that puerarin treatment elevates SOD and GSH-Px while reducing MDA and ROS in bone tissue or serum (Feng, 2018; Chen and Hu, 2021; Yang et al., 2021; Chen, 2024). Mechanistic studies further point toward ROS scavenging as a key mode of action. Xiao et al. (2020) demonstrated that puerarin reduces RANKL-induced intracellular ROS via HO-1 upregulation; silencing of HO-1 partially reversed the suppression of ROS and osteoclast differentiation. Feng and Tang (2024) found that puerarin attenuates FoxO1 phosphorylation-induced inactivation and promotes downstream catalase expression, thereby reducing intracellular ROS in osteoclast precursors and suppressing osteoclast differentiation. Yang et al. (2021) observed that puerarin significantly elevated SOD and GSH-Px activities, decreased MDA, and improved BMD and trabecular architecture in OVX rats. It should be noted, however, that most included studies measured SOD, MDA, and related markers only at the experimental endpoint, that the number of studies reporting these outcomes was small and heterogeneity was high, and that the causal and temporal relationships between oxidative stress amelioration and bone structural recovery have not been experimentally established. Future studies should incorporate time-course sampling designs to delineate the temporal sequence of ROS clearance and bone mass changes.

Collectively, the above mechanistic analysis reveals that the bone-protective effects of puerarin are not dependent on a single target. Oxidative stress and inflammation form a bidirectional positive-feedback loop within the bone microenvironment: ROS activate NF-κB and MAPK to promote pro-inflammatory cytokine transcription, and pro-inflammatory cytokines in turn further increase ROS production through activation of NADPH oxidase and other pathways, creating a vicious cycle that accelerates bone loss (Forrester et al., 2018). Puerarin, on the one hand, scavenges ROS through pathways including HO-1/Nrf2 and FoxO1/catalase, and on the other hand, reduces the levels of pro-inflammatory cytokines such as IL-6 and TNF-α, thereby interrupting this positive-feedback loop at the upstream level. These effects ultimately converge on the suppression of the RANKL/NF-κB/NFATc1 axis, consistent with its overall profile of inhibiting osteoclastogenesis and rectifying high bone turnover. Therefore, the anti-inflammatory and antioxidant effects of puerarin are more appropriately viewed as interconnected components of an integrated bone-protective network rather than as independent pharmacological targets.

### 4.3 Safety of puerarin

Puerarin is generally considered to have a favorable safety profile. Systematic reviews of multiple randomized controlled trials have reported that adverse reactions to puerarin injection are predominantly mild, including facial flushing, dizziness, and nausea, with no serious adverse events documented (Zheng et al., 2017; Xie et al., 2018). Boué et al. (2003) reported that a mixed extract containing puerarin competitively binds ERβ and stimulates MCF-7 cell proliferation, and puerarin was identified as one of the active estrogenic constituents. Saha et al. (2012) found that puerarin, acting as a selective estrogen receptor modulator, blocks implantation and alters endometrial ERα/ERβ expression in rats at high oral doses, suggesting potential risk during early gestation that warrants attention in women of childbearing age. The available toxicological evidence generally indicates low toxicity for puerarin, with no apparent toxicity observed in acute, subacute, and genotoxicity studies (Chung et al., 2009; Liu et al., 2023). Moreover, in preclinical osteoporosis studies, puerarin was administered across a wide range of doses and durations without observable toxicity or mortality (Li et al., 2022; Yang et al., 2026). However, systematic toxicological studies specifically addressing long-term oral puerarin administration with coverage of endometrial and mammary gland endpoints, as well as standardized reproductive and genetic toxicity data, remain lacking. Furthermore, we did not identify any independent prospective cohort studies specifically designed to quantify adverse reactions or hemolysis rates associated with puerarin injectable formulations. Thus, further drug safety investigations are warranted.

### 4.4 Limitations

Although this study adhered to the PRISMA guidelines, several limitations should be acknowledged. (1) All osteoporosis models in the included studies were constructed by ovariectomy, suggesting that puerarin has favorable effects on postmenopausal osteoporosis. Nevertheless, its effectiveness in other forms of osteoporosis remains to be further explored. (2) Despite the demonstrated therapeutic efficacy of puerarin in animal models, inherent pathological differences exist between animal models and human osteoporosis. Consequently, the translational relevance of these preclinical findings to human patients requires validation through future clinical studies. (3) Substantial heterogeneity was observed across the primary outcomes. Although subgroup analyses were performed, the sources of heterogeneity were not fully elucidated, which may introduce uncertainty into the findings. (4) Although a random-effects model was employed for the meta-analysis, variations in measurement methods for outcome indicators may contribute to systematic errors in the pooled estimates. (5) Publication bias was present for the major outcome measures, including BMD and Tb.N. Despite the application of a trim-and-fill correction, the preferential publication of positive results in the original studies may have resulted in an overestimation of the therapeutic effects of puerarin. (6) A portion of the data was extracted from graphs using digitization software (WebPlotDigitizer 4.7), which may have introduced minor measurement error.

## 5 Conclusion

This systematic review and meta-analysis indicates that puerarin improves bone mineral density and bone microarchitecture in female ovariectomized animal models of osteoporosis. The pooled findings are consistent with suppression of bone resorption and normalization of high bone turnover, with possible contributions from anti-inflammatory and antioxidant mechanisms. However, substantial heterogeneity, publication bias, and the exclusive use of ovariectomy models limit the generalizability of the findings. Further rigorously designed preclinical studies are needed before clinical translation can be considered.

## Data Availability

The raw data supporting the conclusions of this article will be made available by the authors, without undue reservation.

## References

[B1] Baird L. Yamamoto M. (2020). The molecular mechanisms regulating the KEAP1-NRF2 pathway. Mol. Cell. Biol. 40, e00099-20. doi: 10.1128/MCB.00099-20 32284348 PMC7296212

[B2] Baron R. Kneissel M. (2013). WNT signaling in bone homeostasis and disease: from human mutations to treatments. Nat. Med. 19, 179–192. doi: 10.1038/nm.3074 23389618

[B3] Bartell S. M. Kim H. N. Ambrogini E. Han L. Iyer S. Serra Ucer S. . (2014). FoxO proteins restrain osteoclastogenesis and bone resorption by attenuating H2O2 accumulation. Nat. Commun. 5, 3773. doi: 10.1038/ncomms4773 24781012 PMC4015330

[B4] Boué S. M. Wiese T. E. Nehls S. Burow M. E. Elliott S. Carter-Wientjes C. H. . (2003). Evaluation of the estrogenic effects of legume extracts containing phytoestrogens. J. Agric. Food. Chem. 51, 2193–2199. doi: 10.1021/jf021114s 12670155

[B5] Capulli M. Paone R. Rucci N. (2014). Osteoblast and osteocyte: games without frontiers. Arch. Biochem. Biophys. 561, 3–12. doi: 10.1016/j.abb.2014.05.003 24832390

[B6] Chen H. R. (2024). Mechanism by which puerarin prevents postmenopausal osteoporosis through Keap1/Nrf2/HO-1 pathway-mediated antioxidant effects. Liaoning University of Traditional Chinese Medicine, Shenyang (China. doi: 10.27213/d.cnki.glnzc.2024.000457

[B7] Chen W. M. Hu X. Y. (2021). Effects of puerarin combined with alendronate sodium on oxidative stress, inflammatory responses, and bone metabolism in rats with postmenopausal osteoporosis. Chin. J. Osteoporosis 27, 73–76. doi: 10.3969/j.issn.1006-7108.2021.01.015

[B8] Chung H. J. Chung M. J. Houng S. J. Jeun J. Kweon D. K. Choi C. H. . (2009). Toxicological evaluation of the isoflavone puerarin and its glycosides. Eur. Food Res. Technol. 230, 145–153. doi: 10.1007/s00217-009-1156-3 30311153

[B9] Compston J. E. McClung M. R. Leslie W. D. (2019). Osteoporosis. Lancet 393, 364–376. doi: 10.1016/S0140-6736(18)32112-3 30696576

[B10] Consensus development conference: diagnosis, prophylaxis, and treatment of osteoporosis (1993). Consensus development conference: diagnosis, prophylaxis, and treatment of osteoporosis. Am. J. Med. 94, 646–650. doi: 10.1016/0002-9343(93)90218-E 8506892

[B11] Domazetovic V. Marcucci G. Iantomasi T. Brandi M. L. Vincenzini M. T. (2017). Oxidative stress in bone remodeling: role of antioxidants. Clin. cases Miner. Bone Metab. 14, 209–216. doi: 10.11138/ccmbm/2017.14.1.209 29263736 PMC5726212

[B12] Eastell R. O’Neill T. W. Hofbauer L. C. Langdahl B. Reid I. R. Gold D. T. . (2016). Postmenopausal osteoporosis. Nat. Rev. Dis. Primers 2, 16069. doi: 10.1038/nrdp.2016.69 27681935

[B13] Feng Y. L. (2018). Mechanisms by which FoxO1 mediates the inhibitory effects of resveratrol and puerarin on osteoclastogenesis and osteoclast activity. Lanzhou University, Lanzhou (China.

[B14] Feng Y. Tang X. (2024). FoxO1 as the critical target of puerarin to inhibit osteoclastogenesis and bone resorption. J. Pharm. Pharmacol. 76, 813–823. doi: 10.1093/jpp/rgae033 38554122

[B15] Forrester S. J. Kikuchi D. S. Hernandes M. S. Xu Q. Griendling K. K. (2018). Reactive oxygen species in metabolic and inflammatory signaling. Circ. Res. 122, 877–902. doi: 10.1161/CIRCRESAHA.117.311401 29700084 PMC5926825

[B16] Huang H. L. Li H. Wang J. H. Li B. Xie J. S. Liu J. (2011). Effects of estrogen combined with different doses of puerarin on osteoporosis in ovariectomized rats. Maternal Child Health Care China 26, 5228–5231.

[B17] Kalyanaraman H. Schall N. Pilz R. B. (2018). Nitric oxide and cyclic GMP functions in bone. Nitric. Oxide 76, 62–70. doi: 10.1016/j.niox.2018.03.007 29550520 PMC9990405

[B18] Kim J. M. Lin C. Stavre Z. Greenblatt M. B. Shim J. H. (2020). Osteoblast-osteoclast communication and bone homeostasis. Cells 9, 2073. doi: 10.3390/cells9092073 32927921 PMC7564526

[B19] Lam J. Takeshita S. Barker J. E. Kanagawa O. Ross F. P. Teitelbaum S. L. (2000). TNF-α induces osteoclastogenesis by direct stimulation of macrophages exposed to permissive levels of RANK ligand. J. Clin. Invest. 106, 1481–1488. doi: 10.1172/JCI11176 11120755 PMC387259

[B20] Lee K. Seo I. Choi M. H. Jeong D. (2018). Roles of mitogen-activated protein kinases in osteoclast biology. Int. J. Mol. Sci. 19, 3004. doi: 10.3390/ijms19103004 30275408 PMC6213329

[B21] Li B. Liu M. Wang Y. Gong S. Yao W. Li W. . (2020). Puerarin improves the bone micro-environment to inhibit OVX-induced osteoporosis via modulating SCFAs released by the gut microbiota and repairing intestinal mucosal integrity. Biomed. Pharmacother. 132, 110923. doi: 10.1016/j.biopha.2020.110923 33125971

[B22] Li B. Wang Y. Gong S. Yao W. Gao H. Liu M. . (2022). Puerarin improves OVX-induced osteoporosis by regulating phospholipid metabolism and biosynthesis of unsaturated fatty acids based on serum metabolomics. Phytomedicine 102, 154198. doi: 10.1016/j.phymed.2022.154198 35636175

[B23] Li Q. Ren H. M. Zhu T. Li W. Y. Bai L. (2021). Effects of puerarin on cytokines and bone metabolism in a rat model of osteoporosis. Chin. J. Osteoporosis 27, 1017–1021. doi: 10.3969/j.issn.1006-7108.2021.07.015

[B24] Li X. J. Zhang C. Y. (2009). Effects of puerarin on bone mineral density and serum IL-6 in ovariectomized rats. J. Changzhi Med. Coll. 23, 13–15. doi: 10.3969/j.issn.1006-0588.2009.01.005 23195197

[B25] Liang H. Kuang Z. Q. Wang P. P. Shen J. Z. Yang L. Zhang R. H. (2012). Puerarin regulates bone metabolism through BMP-2 in ovariectomized rats. J. Guangzhou Med. Univ. 40, 1–4. doi: 10.3969/j.issn.1008-1836.2012.03.01

[B26] Liang Q. Li H. Xie J. S. Huang L. L. Gu J. Z. Li B. . (2019). Effects of puerarin on serum OPG, RANKL, and bone tissue in rats with postmenopausal osteoporosis. Chin. J. Gerontology 39, 4031–4034. doi: 10.3969/j.issn.1005-9202.2019.16.053

[B27] Liu X. Huang R. Wan J. (2023). Puerarin: a potential natural neuroprotective agent for neurological disorders. Biomed. Pharmacother. 162, 114581. doi: 10.1016/j.biopha.2023.114581 36966665

[B28] Lv H. Che T. Tang X. Liu L. Cheng J. (2015). Puerarin enhances proliferation and osteoblastic differentiation of human bone marrow stromal cells via a nitric oxide/cyclic guanosine monophosphate signaling pathway. Mol. Med. Rep. 12, 2283–2290. doi: 10.3892/mmr.2015.3647 25892538

[B29] Lv L. (2006). Mechanisms underlying the effects of plant isoflavones on calcium metabolism in adult ovariectomized rats. Nanjing Normal University, Nanjing (China.

[B30] Lv L. T. Fan W. C. (2023). Effects of puerarin on bone metabolic balance in ovariectomized rats. Western J. Traditional Chin. Med. 36, 27–30. doi: 10.12174/j.issn.2096-9600.2023.07.06

[B31] Marcucci G. Domazetovic V. Nediani C. Ruzzolini J. Favre C. Brandi M. L. (2023). Oxidative stress and natural antioxidants in osteoporosis: novel preventive and therapeutic approaches. Antioxidants 12, 373. doi: 10.3390/antiox12020373 36829932 PMC9952369

[B32] McGregor N. E. Murat M. Elango J. Poulton I. J. Walker E. C. Crimeen-Irwin B. . (2019). IL-6 exhibits both cis- and trans-signaling in osteocytes and osteoblasts, but only trans-signaling promotes bone formation and osteoclastogenesis. J. Biol. Chem. 294, 7850–7863. doi: 10.1074/jbc.RA119.008074 30923130 PMC6514630

[B33] Montaseri A. Giampietri C. Rossi M. Riccioli A. Del Fattore A. Filippini A. (2020). The role of autophagy in osteoclast differentiation and bone resorption function. Biomolecules 10, 1398. doi: 10.3390/biom10101398 33008140 PMC7601508

[B34] Oei L. Koromani F. Breda S. J. Schousboe J. T. Clark E. M. van Meurs J. B. . (2018). Osteoporotic vertebral fracture prevalence varies widely between qualitative and quantitative radiological assessment methods: the Rotterdam Study. J. Bone Miner. Res. 33, 560–568. doi: 10.1002/jbmr.3220 28719143

[B35] Papapetrou P. D. (2009). Bisphosphonate-associated adverse events. Hormones 8, 96–110. doi: 10.14310/horm.2002.1226 19570737

[B36] Park J. H. Lee N. K. Lee S. Y. (2017). Current understanding of RANK signaling in osteoclast differentiation and maturation. Mol. Cells 40, 706–713. doi: 10.14348/molcells.2017.0225 29047262 PMC5682248

[B37] Perrone P. De Rosa C. D’Angelo S. (2025). Polyphenols and bone health: a comprehensive review of their role in osteoporosis prevention and treatment. Molecules 30, 4154. doi: 10.3390/molecules30214154 41226117 PMC12608581

[B38] Ponzetti M. Rucci N. (2019). Updates on osteoimmunology: what’s new on the cross-talk between bone and immune system. Front. Endocrinol. 10, 236. doi: 10.3389/fendo.2019.00236 31057482 PMC6482259

[B39] Ponzetti M. Rucci N. (2021). Osteoblast differentiation and signaling: established concepts and emerging topics. Int. J. Mol. Sci. 22, 6651. doi: 10.3390/ijms22136651 34206294 PMC8268587

[B40] Qiu Z. Li L. Huang Y. Shi K. Zhang L. Huang C. . (2022). Puerarin specifically disrupts osteoclast activation via blocking integrin-β3 Pyk2/Src/Cbl signaling pathway. J. Orthop. Translat. 33, 55–69. doi: 10.1016/j.jot.2022.01.003 35228997 PMC8858883

[B41] Reid I. R. (2015). Efficacy, effectiveness and side effects of medications used to prevent fractures. J. Intern. Med. 277, 690–706. doi: 10.1111/joim.12339 25495429

[B42] Ruan H. Li W. Chang H. Wen M. Luo S. Song F. . (2025). Antioxidant, hypoglycemic, and hypolipidemic effects of puerarin *in vivo*. Food Sci. Nutr. 13, e70257. doi: 10.1002/fsn3.70257 40336535 PMC12056237

[B43] Saha P. Saraswat G. Chakraborty P. Banerjee S. Pal B. C. Kabir S. N. (2012). Puerarin, a selective oestrogen receptor modulator, disrupts pregnancy in rats at pre-implantation stage. Reproduction 144, 633–645. doi: 10.1530/REP-11-0423 22919047

[B44] Salari N. Ghasemi H. Mohammadi L. Behzadi M. H. Rabieenia E. Shohaimi S. . (2021). The global prevalence of osteoporosis in the world: a comprehensive systematic review and meta-analysis. J. Orthop. Surg. Res. 16, 609. doi: 10.1186/s13018-021-02772-0 34657598 PMC8522202

[B45] Sarafrazi N. Wambogo E. A. Shepherd J. A. (2021). Osteoporosis or low bone mass in older adults: United States 2017–2018. NCHS Data Brief 405, 1–8. doi: 10.15620/cdc:103477 34029181

[B46] Takito J. Inoue S. Nakamura M. (2018). The sealing zone in osteoclasts: a self-organized structure on the bone. Int. J. Mol. Sci. 19, 984. doi: 10.3390/ijms19040984 29587415 PMC5979552

[B47] Tang X. L. (2005). Oxidative stress status and osteoporosis in ovariectomized rats: the intervention effect of puerarin. Lanzhou University, Lanzhou (China.

[B48] Tang W. Xiao L. Ge G. Zhong M. Zhu J. Qin J. . (2020). Puerarin inhibits titanium particle-induced osteolysis and RANKL-induced osteoclastogenesis via suppression of the NF-κB signaling pathway. J. Cell. Mol. Med. 24, 11972–11983. doi: 10.1111/jcmm.15821 32896108 PMC7578865

[B49] Wang L. T. Chen L. R. Chen K. H. (2023). Hormone-related and drug-induced osteoporosis: a cellular and molecular overview. Int. J. Mol. Sci. 24, 5814. doi: 10.3390/ijms24065814 36982891 PMC10054048

[B50] Wang P. P. Zhu X. F. Yang L. Liang H. Feng S. W. Zhang R. H. (2012). Puerarin stimulates osteoblasts differentiation and bone formation through estrogen receptor, p38 MAPK, and Wnt/β-catenin pathways. J. Asian Nat. Prod. Res. 14, 897–905. doi: 10.1080/10286020.2012.702757 22917468

[B51] Wang Z. H. Lin Z. Y. (2019). Experimental study of puerarin combined with zinc in the treatment of osteoporosis in ovariectomized rats. Chin. J. Gen. Pract. 17, 1450–1453. doi: 10.16766/j.cnki.issn.1674-4152.000967

[B52] Weitzmann M. N. Ofotokun I. (2016). Physiological and pathophysiological bone turnover: role of the immune system. Nat. Rev. Endocrinol. 12, 518–532. doi: 10.1038/nrendo.2016.91 27312863 PMC5857945

[B53] Wen L. Wang L. W. Wang T. Zhang W. L. (2025). Effects of puerarin on regulation of the canonical Wnt signaling pathway in osteoporotic rats. Acta Neuropharmacologica 15, 1–6. doi: 10.3969/j.issn.2095-1396.2025.03.001

[B54] Wu M. Chen G. Li Y. P. (2016). TGF-β and BMP signaling in osteoblast, skeletal development, and bone formation, homeostasis and disease. Bone Res. 4, 16009. doi: 10.1038/boneres.2016.9 27563484 PMC4985055

[B55] Wu M. Wu S. Chen W. Li Y. P. (2024). The roles and regulatory mechanisms of TGF-β and BMP signaling in bone and cartilage development, homeostasis and disease. Cell Res. 34, 101–123. doi: 10.1038/s41422-023-00918-9 38267638 PMC10837209

[B56] Xiao L. Zhong M. Huang Y. Zhu J. Tang W. Li D. . (2020). Puerarin alleviates osteoporosis in the ovariectomy-induced mice by suppressing osteoclastogenesis via inhibition of TRAF6/ROS-dependent MAPK/NF-κB signaling pathways. Aging 12, 21706–21729. doi: 10.18632/aging.103976 33176281 PMC7695364

[B57] Xie B. Wang Q. Zhou C. Wu J. Xu D. (2018). Efficacy and safety of the injection of the traditional Chinese medicine puerarin for the treatment of diabetic peripheral neuropathy: a systematic review and meta-analysis of 53 randomized controlled trials. Evid. Based Complement. Alternat. Med. 2018, 2834650. doi: 10.1155/2018/2834650 29619066 PMC5830025

[B58] Xu X. S. Pang Z. B. Yang J. Zhou Y. F. Xia C. J. Ding X. Y. (2021). Effects of puerarin on bone loss and the RANKL/RANK/OPG signaling pathway in ovariectomized mice. Zhejiang Med. J. 43, 471–474. doi: 10.12056/j.issn.1006-2785.2021.43.5.2020-2134

[B59] Yang D. Guan Z. Y. (2023). Effects of puerarin in ovariectomized osteoporotic rats through the PPAR-γ/Axin2/Wnt signaling pathway. Chin. J. Gerontology 43, 3228–3232. doi: 10.3969/j.issn.1005-9202.2023.13.041

[B60] Yang Z. H. Hao L. S. Zhang J. X. (2021). Effects of puerarin on oxidative stress, bone metabolism, and bone mineral density in osteoporotic rats. Chin. J. Osteoporosis 27, 413–417. doi: 10.3969/j.issn.1006-7108.2021.03.020

[B61] Yang Z. Tang R. Ma Y. Luo W. Hu Y. (2026). The anti-osteoporotic effect of puerarin on the femoral bone in rat models of osteoporosis: a systematic review and meta-analysis. Front. Pharmacol. 16, 1712682. doi: 10.3389/fphar.2025.1712682 41560734 PMC12813136

[B62] Yao Y. Cai X. Chen Y. Zhang M. Zheng C. (2025). Estrogen deficiency-mediated osteoimmunity in postmenopausal osteoporosis. Med. Res. Rev. 45, 561–575. doi: 10.1002/med.22081 39234932

[B63] Yu S. Ji Q. H. Wang J. Liu Y. C. Mi X. L. Yang S. Y. (2019). Experimental study of the therapeutic effects and mechanisms of puerarin combined with estrogen in rats with postmenopausal osteoporosis. Heilongjiang Med. Pharm. 42, 213–215. doi: 10.3969/j.issn.1008-0104.2019.04.103

[B64] Yue H. Z. Cai J. Ma X. Q. Wang Y. (2021). Effects of puerarin on bone metabolism, bone mineral density, and bone biomechanics in rats with postmenopausal osteoporosis. Chin. J. Osteoporosis 27, 77–81. doi: 10.3969/j.issn.1006-7108.2021.01.016

[B65] Zeng J. Zhao N. Yang J. Kuang W. Xia X. Chen X. . (2022). Puerarin induces molecular details of ferroptosis-associated anti-inflammatory on RAW264.7 macrophages. Metabolites 12, 653. doi: 10.3390/metabo12070653 35888777 PMC9317776

[B66] Zeng S. L. Shi X. B. (2018). Effects of puerarin on osteoporosis and the PI3K/Akt signaling pathway in ovariectomized female rats. Hebei Med. J. 40, 3566–3569. doi: 10.3969/j.issn.1002-7386.2018.23.010

[B67] Zhang C. Li H. Li J. Hu J. Yang K. Tao L. (2023). Oxidative stress: a common pathological state in a high-risk population for osteoporosis. Biomed. Pharmacother. 163, 114834. doi: 10.1016/j.biopha.2023.114834 37163779

[B68] Zhang G. Wang Y. Tang G. Ma Y. (2019). Puerarin inhibits the osteoclastogenesis by inhibiting RANKL-dependent and -independent autophagic responses. BMC Complement. Altern. Med. 19, 269. doi: 10.1186/s12906-019-2691-5 31615565 PMC6794871

[B69] Zhang P. Lin L. Wang N. Wang Y. Miao Y. Fei J. . (2025). Yougui pills prevent ovariectomy-induced bone loss by suppressing Th17 response and IL-17/NF-κB pathway. Ann. Med. 57, 2529576. doi: 10.1080/07853890.2025.2529576 40622369 PMC12239122

[B70] Zhang P. Ye J. Dai J. Wang Y. Chen G. Hu J. . (2022). Gallic acid inhibits osteoclastogenesis and prevents ovariectomy-induced bone loss. Front. Endocrinol. 13, 963237. doi: 10.3389/fendo.2022.963237 36601012 PMC9807166

[B71] Zhao X. Zhou J. Liu Y. Wang J. Liu Y. Wang B. . (2024). Puerarin alleviates osteoporosis in rats by targeting the JAK2/STAT3 signaling pathway. Biomol. Biomed. 24, 1651–1661. doi: 10.17305/bb.2024.10500 38843496 PMC11496846

[B72] Zheng Q. H. Li X. L. Mei Z. G. Xiong L. Mei Q. X. Wang J. F. . (2017). Efficacy and safety of puerarin injection in curing acute ischemic stroke: a meta-analysis of randomized controlled trials. Medicine 96, e5803. doi: 10.1097/MD.0000000000005803 28072733 PMC5228693

[B73] Zhou J. X. Wang B. Y. Zhao X. L. Zhang Y. J. (2025). Mechanism of puerarin in osteoporotic rats based on the miR-21-5p/TGF-β1/Smads signaling pathway. Chin. Pharm. J. 60, 1923–1930. doi: 10.11669/cpj.2025.18.006

[B74] Zhu X. Bai W. Zheng H. (2021). Twelve years of GWAS discoveries for osteoporosis and related traits: advances, challenges and applications. Bone Res. 9, 23. doi: 10.1038/s41413-021-00143-3 33927194 PMC8085014

