# Supplementary material for: Protective effects and mechanisms of puerarin in ovariectomized animal models of osteoporosis: a systematic review and meta-analysis of preclinical studies
Source: Front Physiol. 2026 Aug 27;17:1911109. doi: 10.3389/fphys.2026.1911109 (PMC13558411; doi:10.3389/fphys.2026.1911109)
Supplement: Supplementary file 1 [file SupplementaryFile1.docx]

**Supplementary Table 1**. Search strategy of each database.

| **Databases** | **Search strategies** |
| --- | --- |
| **PubMed** | (("Osteoporosis"[Mesh]) OR (((((((((((((((((((((Osteoporoses[Title/Abstract]) OR (Osteoporosis, Age-Related[Title/Abstract])) OR (Osteoporosis, Age Related[Title/Abstract])) OR (Age-Related Osteoporosis[Title/Abstract])) OR (Age-Related Osteoporoses[Title/Abstract])) OR (Age Related Osteoporosis[Title/Abstract])) OR (Osteoporoses, Age-Related[Title/Abstract])) OR (Age-Related Bone Loss[Title/Abstract])) OR (Age-Related Bone Losses[Title/Abstract])) OR (Bone Loss, Age-Related[Title/Abstract])) OR (Bone Loss, Age Related[Title/Abstract])) OR (Bone Losses, Age-Related[Title/Abstract])) OR (Osteoporosis, Senile[Title/Abstract])) OR (Osteoporoses, Senile[Title/Abstract])) OR (Senile Osteoporoses[Title/Abstract])) OR (Senile Osteoporosis[Title/Abstract])) OR (Osteoporosis, Involutional[Title/Abstract])) OR (Osteoporosis, Post-Traumatic[Title/Abstract])) OR (Osteoporosis, Post Traumatic[Title/Abstract])) OR (Post-Traumatic Osteoporoses[Title/Abstract])) OR (Post-Traumatic Osteoporosis[Title/Abstract]))) AND ((("puerarin"[Title/Abstract]) OR (kakonein[Title/Abstract])) OR (7,4'-dihydroxyisoflavone-8beta-glucopyranoside[Title/Abstract])) |
| **Web of science** | #3 #1 AND #2  #2 puerarin (Topic) or kakonein (Topic) or 7,4'-dihydroxyisoflavone-8beta-glucopyranoside (Topic)  #1 Osteoporosis (Topic) or Osteoporoses (Topic) or Osteoporosis, Age-Related (Topic) or Osteoporosis, Age Related (Topic) or Osteoporoses, Age-Related (Topic) or Age-Related Osteoporosis (Topic) or Age-Related Osteoporoses (Topic) or Age Related Osteoporosis (Topic) or Age-Related Bone Loss (Topic) or Age-Related Bone Losses (Topic) or Bone Loss, Age-Related (Topic) or Bone Loss, Age Related (Topic) or Bone Losses, Age-Related (Topic) or Osteoporosis, Senile (Topic) or Osteoporoses, Senile (Topic) or Senile Osteoporoses (Topic) or Senile Osteoporosis (Topic) or Osteoporosis, Involutional (Topic) or Osteoporosis, Post-Traumatic (Topic) or Osteoporosis, Post Traumatic (Topic) or Post-Traumatic Osteoporoses (Topic) or Post-Traumatic Osteoporosis (Topic) |
| **Embase** | #5 #1 AND #4  #4 #2 OR #3  #3 'osteoporoses':ab,ti OR 'osteoporosis, age-related':ab,ti OR 'osteoporosis, age related':ab,ti OR 'osteoporoses, age-related':ab,ti OR 'age-related osteoporosis':ab,ti OR 'age related osteoporosis':ab,ti OR 'age-related osteoporoses':ab,ti OR 'age-related bone loss':ab,ti OR 'age-related bone losses':ab,ti OR 'bone loss, age-related':ab,ti OR 'bone loss, age related':ab,ti OR 'bone losses, age-related':ab,ti OR 'osteoporosis, senile':ab,ti OR 'osteoporoses, senile':ab,ti OR 'senile osteoporoses':ab,ti OR 'senile osteoporosis':ab,ti OR 'osteoporosis, Involutional':ab,ti OR 'osteoporosis, post-traumatic':ab,ti OR 'osteoporosis, post traumatic':ab,ti OR 'post-traumatic osteoporoses':ab,ti OR 'post-traumatic osteoporosis':ab,ti  #2 'osteoporosis'/exp  #1 'puerarin'/exp |
| **CBM** | 7 (#6) OR (#5)  6 (#4) OR (#3)  5 (#2) OR (#1)  4 "葛根黄素"[常用字段:智能]  3 "葛根素"[不加权:扩展]  2 "年龄相关骨质疏松"[常用字段:智能] OR "年龄相关骨质丢失"[常用字段:智能] OR "骨质疏松症"[常用字段:智能] AND "骨质疏松, 创伤后"[常用字段:智能] OR " 创伤后骨质疏松"[常用字段:智能] OR "创伤后-骨质疏松"[常用字段:智能] OR "骨质疏松, 老年性"[常用字段:智能] OR "老年性骨质疏松"[常用字段:智能] OR "骨质疏松, 年龄相关"[常用字段:智能]  1 "骨质疏松"[不加权:扩展] |
| **CNKI** | SU%=('骨质疏松' + '骨质疏松症' + '骨质流失' + '骨质丢失' + '骨密度' + '骨代谢') * ('葛根素' + '葛根黄素') |
| **WanFang** | 主题: (("骨质疏松" or "骨质疏松症" or "骨质流失" or "骨质丢失" or "骨密度" or "骨代谢") and ("葛根素" or "葛根黄素")) |
| **VIP** | M=((骨质疏松 OR 骨质疏松症 OR 骨质流失 OR 骨质丢失 OR 骨密度 OR 骨代谢) AND (葛根素 OR 葛根黄素)) |

**Supplementary Table 2**. Manufacturer and batch number of puerarin

| Author, year | Manufacturer and batch number of puerarin |
| --- | --- |
| Chen 2021 | Ankang Beiyida Pharmaceutical Co., Ltd., National Drug Approval Number: H20053739 |
| Chen 2024 | Sigma, USA, purity ≥98% |
| Feng 2018 | Dalian Meilun Biotechnology Co., Ltd. |
| Huang 2011 | Chengdu Tiantai Pharmaceutical Factory, batch number: 0905053 |
| Li 2009 | National Institute for the Control of Pharmaceutical and Biological Products, China |
| Li 2020 | J&K Scientific Ltd (Beijing, China) , purity >98% |
| Li 2021 | Guangdong Xinfeng Pharmaceutical Co., Ltd., National Drug Approval Number: H20044953 |
| Liang 2012 | Guizhou Dida Company, production batch number: 20100820, purity 99% |
| Lv 2006 | Self-extracted, content: 89.69% |
| Lv 2023 | Baizheng Pharmaceutical, National Drug Approval Number: H20054812 |
| Tang 2005 | Guangdong Yantang Biochemical Pharmaceutical Co., Ltd., batch number: 0110181 |
| Wang 2019 | Sigma |
| Wen 2025 | Qingdao Yunshan Biotechnology Co., Ltd. |
| Xiao 2020 | Sigma-Aldrich, Sydney, Australia |
| Xu 2021 | Not mentioned |
| Yang 2021 | Shaanxi Ankang Pharmaceutical Factory, production batch number: 1902068 |
| Yang 2023 | Ankang Beiyida Pharmaceutical Co., Ltd., National Drug Approval Number: H20053739 |
| Yu 2019 | Heilongjiang Jiangshi Pharmaceutical Co., Ltd., National Drug Approval Number: H20043845 |
| Yue 2021 | Shaanxi Ankang Pharmaceutical Factory |
| Zeng 2018 | Hubei Jusheng Technology Co., Ltd., batch number: 20160806 |
| Zhao 2024 | Zhejiang Zhenyuan Pharmaceutical Co., Ltd., China, National Drug Approval Number: H20020392, purity >99% |
| Zhou 2025 | Hubei Cuichuang Biotechnology Co., Ltd., batch number: 117076-54-5, purity ≥98% |

**Supplementary Table 3**. Subgroup analysis of primary outcome indicators.

| **Variables** | **SMD [95%CI]** | ***P* value** | | ***I^2^*(%)** | ***P*-heterogeneity** |
| --- | --- | --- | --- | --- | --- |
| **BMD** |  |  |  | |  |
| dose |  |  |  | |  |
| ≤50 mg/kg | 2.49 [1.69, 3.30] | 0.000 | 82.7 | | 0.000 |
| >50 mg/kg | 2.84 [1.94, 3.75] | 0.000 | 73.9 | | 0.000 |
| route |  |  |  | |  |
| gavage | 3.21 [1.98, 4.44] | 0.000 | 84.7 | | 0.000 |
| intraperitoneal injection | 3.67 [2.89, 4.45] | 0.000 | 0.0 | | 0.527 |
| subcutaneous injection | 1.92 [1.28, 2.56] | 0.000 | 68.4 | | 0.001 |
| duration |  |  |  | |  |
| <8 weeks | 2.52 [1.69, 3.34] | 0.000 | 76.6 | | 0.000 |
| 8≤t＜12 weeks | 2.63 [1.71, 3.54] | 0.000 | 69.2 | | 0.006 |
| ≥12 weeks | 2.97 [1.41, 4.53] | 0.000 | 87.8 | | 0.000 |
| species |  |  |  | |  |
| rats | 2.61 [1.97, 3.26] | 0.000 | 81.2 | | 0.000 |
| mice | 2.62 [2.02, 3.22] | 0.000 | 79.6 | | 0.000 |
| **Tb.N** |  |  |  | |  |
| dose |  |  |  | |  |
| ≤50 mg/kg | 3.90 [0.55, 7.24] | 0.022 | 95.4 | | 0.000 |
| >50 mg/kg | 3.39 [1.92, 4.85] | 0.000 | 86.8 | | 0.000 |
| route |  |  |  | |  |
| gavage | 3.03 [1.13, 4.93] | 0.002 | 90.9 | | 0.000 |
| intraperitoneal injection | 4.01 [3.04, 4.98] | 0.000 | 0.0 | | 0.592 |
| subcutaneous injection | 4.08 [1.02, 7.15] | 0.009 | 92.2 | | 0.000 |
| duration |  |  |  | |  |
| <8 weeks | 5.21 [3.02, 7.41] | 0.011 | 77.8 | | 0.011 |
| 8≤t＜12 weeks | 1.92 [0.86, 2.98] | 0.011 | 72.9 | | 0.011 |
| ≥12 weeks | 4.62 [0.34, 8.89] | 0.000 | 94.6 | | 0.000 |
| species |  |  |  | |  |
| rats | 3.63 [2.06, 5.20] | 0.000 | 91.9 | | 0.000 |
| mice | 3.27 [1.21, 5.33] | 0.002 | 62.3 | | 0.103 |
| **Tb.Sp** |  |  |  | |  |
| dose |  |  |  | |  |
| ≤50 mg/kg | -4.53 [-8.97, -0.09] | 0.046 | 96.7 | | 0.000 |
| >50 mg/kg | -3.76 [-5.64, -1.88] | 0.000 | 88.3 | | 0.000 |
| route |  |  |  | |  |
| gavage | -3.66 [-6.32, -1.01] | 0.007 | 93.1 | | 0.000 |
| intraperitoneal injection | -5.49 [-6.87, -4.11] | 0.000 | - | | - |
| subcutaneous injection | -4.18 [-7.13, -1.23] | 0.005 | 91.5 | | 0.000 |
| duration |  |  |  | |  |
| <8 weeks | -6.51 [-8.80, -4.23] | 0.000 | 68.8 | | 0.073 |
| 8≤t＜12 weeks | -1.98 [-2.94, -1.03] | 0.000 | 54.7 | | 0.110 |
| ≥12 weeks | -4.79 [-9.78, -0.21] | 0.060 | 95.3 | | 0.000 |
| species |  |  |  | |  |
| rats | -4.55 [-6.63, -2.48] | 0.000 | 93.1 | | 0.000 |
| mice | -1.24 [-2.21, -0.28] | 0.012 | - | | - |
| **Tb.Th** |  |  |  | |  |
| dose |  |  |  | |  |
| ≤50 mg/kg | 3.88 [0.74, 7.01] | 0.015 | 95.4 | | 0.000 |
| >50 mg/kg | 2.46 [1.03, 3.89] | 0.001 | 88.2 | | 0.000 |
| route |  |  |  | |  |
| gavage | 1.86 [0.39, 3.34] | 0.013 | 88.7 | | 0.000 |
| intraperitoneal injection | 2.44 [1.61, 3.26] | 0.000 | - | | - |
| subcutaneous injection | 4.91 [1.74, 8.09] | 0.002 | 20.2 | | 0.263 |
| duration |  |  |  | |  |
| <8 weeks | 5.96 [-1.16, 13.07] | 0.101 | 96.2 | | 0.000 |
| 8≤t＜12 weeks | 2.80 [0.56, 5.04] | 0.014 | 91.8 | | 0.000 |
| ≥12 weeks | 1.44 [0.15, 2.72] | 0.029 | 78.6 | | 0.009 |
| species |  |  |  | |  |
| rats | 3.22 [1.90, 4.53] | 0.000 | 89.4 | | 0.000 |
| mice | 0 [-0.88, 0.88] | 1 | - | | - |
| BV/TV |  |  |  | |  |
| dose |  |  |  | |  |
| ≤50 mg/kg | 2.92 [-0.47, 6.31] | 0.092 | 95.4 | | 0.000 |
| >50 mg/kg | 3.43 [2.00, 4.86] | 0.000 | 85.3 | | 0.000 |
| route |  |  |  | |  |
| gavage | 3.01 [1.02, 5.01] | 0.000 | 88.9 | | 0.000 |
| intraperitoneal injection | 6.49 [2.13, 10.85] | 0.004 | 75.9 | | 0.042 |
| subcutaneous injection | 2.25 [0.94, 3.56] | 0.001 | 76.1 | | 0.015 |
| duration |  |  |  | |  |
| <8 weeks | 4.46 [1.09, 7.84] | 0.010 | 93.8 | | 0.000 |
| 8≤t＜12 weeks | 3.66 [2.25, 5.06] | 0.000 | 71.3 | | 0.015 |
| ≥12 weeks | 1.63 [-0.45, 3.71] | 0.124 | 86.3 | | 0.007 |
| species |  |  |  | |  |
| rats | 2.86 [1.61, 4.12] | 0.000 | 87.8 | | 0.000 |
| mice | 6.03 [0.49, 11.56] | 0.033 | 84.1 | | 0.012 |

**Supplementary Table 4**. Results from trim-and-fill analysis.

| **Variables** | **No. of Trials** | **SMD [95%CI]** | ***P* value** |
| --- | --- | --- | --- |
| **BMD** |  |  |  |
| before trim and fill | 21 | 2.62 [2.02, 3.22] | 0.000 |
| after trim and fill | 28 | 5.86 [3.02, 11.35] | 0.000 |
| **Tb.N** |  |  |  |
| before trim and fill | 10 | 3.55 [2.23, 4.86] | 0.000 |
| after trim and fill | 14 | 6.99 [1.77, 27.65] | 0.000 |

**Supplementary Table 5**. Subgroup analysis of secondary outcome measures

| **Variables** | **SMD [95%CI]** | ***P* value** | | ***I^2^*(%)** | ***P*-heterogeneity** |
| --- | --- | --- | --- | --- | --- |
| BGP |  |  |  | |  |
| route |  |  |  | |  |
| gavage | 0.19 [-2.34, 2.72] | 0.884 | 92.6 | | 0.000 |
| intraperitoneal injection | -3.32 [-4.72, -1.93] | 0.000 | - | | - |
| subcutaneous injection | -2.80 [-4.23, -1.37] | 0.000 | 80.7 | | 0.001 |
| duration |  |  |  | |  |
| <8 weeks | -2.81 [-4.25, -1.37] | 0.000 | 80.9 | | 0.001 |
| 8≤t＜12 weeks | -2.12 [-4.24, -0.00] | 0.050 | 84.5 | | 0.011 |
| ≥12 weeks | 1.48 [0.48, 2.49] | 0.004 | - | | - |
| ALP |  |  |  | |  |
| route |  |  |  | |  |
| gavage | -0.02 [-2.94, 2.91] | 0.991 | 94.7 | | 0.000 |
| intraperitoneal injection | -3.23 [-4.09, -2.36] | 0.000 | 15.8 | | 0.276 |
| subcutaneous injection | -1.95 [-2.73, -1.16] | 0.000 | 0.0 | | 0.528 |
| duration |  |  |  | |  |
| <8 weeks | -2.68 [-3.81, -1.55] | 0.000 | 67.6 | | 0.046 |
| 8≤t＜12 weeks | -2.64 [-3.62, -1.66] | 0.000 | 19.2 | | 0.266 |
| ≥12 weeks | 1.51 [-0.43, 3.44] | 0.128 | 83.8 | | 0.013 |
| **TRACP-5b** |  |  |  | |  |
| dose |  |  |  | |  |
| ≤50 mg/kg | -2.41 [-3.58, -1.25] | 0.000 | 79.5 | | 0.001 |
| >50 mg/kg | -2.53 [-4.00, -1.05] | 0.001 | 78.4 | | 0.010 |
| route |  |  |  | |  |
| gavage | -3.13 [-5.96, -0.30] | 0.030 | 86.9 | | 0.006 |
| intraperitoneal injection | -5.46 [-7.45, -3.47] | 0.000 | - | | - |
| subcutaneous injection | -1.76 [-2.35, -1.18] | 0.000 | 39.2 | | 0.160 |
| duration |  |  |  | |  |
| <8 weeks | -2.30 [-3.66, -0.94] | 0.001 | 82.0 | | 0.001 |
| 8≤t＜12 weeks | -2.03 [-2.74, -1.32] | 0.000 | 24.9 | | 0.264 |
| ≥12 weeks | -4.66 [-6.42, -2.91] | 0.000 | - | | - |
| species |  |  |  | |  |
| rats | -2.56 [-3.54, -1.58] | 0.000 | 79.1 | | 0.000 |
| mice | -1.77 [-2.82, -0.72] | 0.001 | - | | - |

Supplementary Figure 1. Sensitivity analysis plot for the primary outcome measure.

A BMD; B Tb.N; C Tb.Sp; D Tb.Th; E BV/TV; F Conn.D
